# Effect of Ultrasound-Assisted Extraction of Carotenoids from Papaya (*Carica papaya* L. cv. Sweet Mary) Using Vegetable Oils

**DOI:** 10.3390/molecules27030638

**Published:** 2022-01-19

**Authors:** Sara Lara-Abia, Jorge Welti-Chanes, M. Pilar Cano

**Affiliations:** 1 Department of Biotechnology and Food Microbiology, Institute of Food Science Research (CIAL) (CSIC-UAM), 28001 Madrid, Spain; sara.lara.abia@gmail.com; 2 Tecnologico de Monterrey, Escuela de Ingeniería y Ciencias, Monterrey 64000, Mexico; jwelti@tec.mx

**Keywords:** ultrasound-assisted extraction (UAE), green solvents, vegetable oils, carotenoids, carotenoid esters, *Carica papaya* fruit by-products

## Abstract

By-products from fruits and are of great interest for their potential use in the food industry due to their high content of bioactive compounds. Herein, we examined the ultrasound-assisted extraction (UAE) of carotenoid and carotenoid esters from papaya pulp and peel using soybean oil and sunflower oil as alternative green solvents. Response surface methodology (RSM) was established to optimize the UAE process. Three independent variables, ultrasonic amplitude (20–60%), time (10–60 min), and co-solvent percentage (ethanol) (5–20%, *v*/*v*), were applied. The highest total carotenoid content in the UAE extracts was obtained from papaya pulp extracts (58.7 ± 1.6 and 56.0 ± 1.5 μg carotenoids/g oil) using soybean oil and sunflower oil, respectively (60% amplitude/ 10 min/ 20% ethanol). On the other hand, the highest carotenoid content (52.0 ± 0.9 μg carotenoids/g oil) was obtained from papaya peel using soybean oil applying the UAE process (20% amplitude/ 77 min/ 20% ethanol); a minor content of 39.3 ± 0.5 μg carotenoids/g oil was obtained from papaya peel using sunflower oil at 60% amplitude/ 60 min/ 5% ethanol. Lycopene was the most abundant carotenoid among all individual carotenoids observed in papaya oil extracts, obtaining the highest yields of this carotenoid when papaya pulp and peel were extracted using soybean oil (94% and 81%, respectively) and sunflower oil (95% and 82%, respectively). Great extraction of xanthophyll esters was detected using 20% of ethanol in the vegetable oil extraction solvent (*v*/*v*). High correlations (>0.85) was obtained between total carotenoid content and color determination in the UAE oil extracts. UAE vegetable oil extracts enriched with carotenoids from papaya by-products could be useful to formulate new food ingredients based on emulsions with interesting potential health benefits.

## 1. Introduction

Papaya (*Carica papaya* L.) is a tropical fruit-bearing tree with origins in southern Mexico to Colombia and Venezuela; nowadays this fruit is grown widely in tropical and subtropical regions around the world (Latin America, India, and Africa, among others). According to the last data collected by FAO (2020) [1], India is the top papaya producer worldwide (6 MT), representing 50% of world papaya production; however, this production is mainly destined to its internal market. On the other hand, Mexico is registered as the main exporter in the world (participation volume of 47%), with an annual production of 1 million 118 thousand tons, and the United States has been registered as the principal importer (with a quote of 56% of the volume). Although papaya cv. Maradol is the most popular variety worldwide, new cultivars of papaya (Sweet Mary, Alicia, and Eksotika) are set in the Canary Islands (Spain). Among these new varieties, papaya cv. Sweet Mary is an interesting variety to investigate due to its high concentration in bioactive compounds and to promote national production and commercialization. Currently, Spain is the largest papaya producer in Europe (annual production of 15,000 tons, approximately) and the Canary Islands are the largest producers (farms totaling 300 ha) [2,3]. Papaya is a climacteric fruit, in which maturation occurs very sudden after recollection. Its short postharvest life leads to 30–70% of papaya losses. Papaya commercial production and consumption are very extensive worldwide, which lead to large generation of by-products; approx. 50% of papaya fruit is used, while the rest is wasted [4,5]. The generation of such waste turns papaya whole fruit by-products (pulp and peel) into great primary materials to obtain bioactive compounds, which promote health benefits, such as carotenoids, giving an added value to these papaya by-products.

Traditionally, papaya fruits have had different usages depending on the need, e.g., papaya root juice has been used to treat boils, burns, and warts [6]; papaya fruits and leaves contain protein-digesting enzymes such as papain and chymopapain, which have proven their effect on injured skin tissue, removing damaged tissues from wounds and limiting infections [7,8,9]. A recent study reported by Logozzi et al. [10], showed that fermented papaya preparations (specifically the commercialized product FPP^®^) induced increase in telomeres length in bone marrow and ovary, together with an increase in the plasmatic levels of telomerase activity and antioxidant levels, with an efficient decrease in radical oxygen species (ROS). Although these findings are encouraging, additional research is needed. Papaya tissues (pulp and peel) and seeds extracts contain vitamins, bioactive compounds, and a lipid composition that may have positive benefits to human health such as preventing chronic diseases (cancer and cardiovascular diseases), antifungal, antiparasitic, blood lipid-lowering, and lowering sugar levels in blood [11,12]. Additionally, papaya fruit has shown strong antioxidant activity due to its high concentration in pro-vitamin A carotenoids, i.e., β-cryptoxanthin, α-carotene, and β-carotene [13].

As detected gradually over the years, the global penchant for plant derived natural ingredients has led researchers to focus their investigations on the isolation of bioactive compounds from vegetal and fruit wastes involving green processes covering, in this way, consumers’ demand for less processed and/or natural products while maintaining an ecological imbalance supporting green consumerism. Green extraction technologies, i.e., ultrasound-assisted extraction (UAE), microwave-assisted extraction (MAE), or high hydrostatic pressure-assisted extraction (HHPAE), among others, revalorize food wastes and by-products, minimizing the use of organic solvents (petro-based chemicals), reducing the consumption of energy, bringing forth safer products for consumption while being more eco-friendly for the environment. Hoover [14] defined ultrasound as an energy form that travels thought out sound waves ≥ 20,000 vibrations per second. Mason [15] established the ultrasound as any sound with a frequency higher than the human ear is able to perceive (16 KHz). There are two types of intensity application: low ultrasonic intensity (MHz) (aprox. 0.1 W/cm^2^), which it is applied when the objective is to obtain information about the environment (diagnosis) without promoting any modification on its state, and high ultrasonic intensity (KHz) (aprox. 1 W/cm^2^), which it is used to perform permanent changes in the treated medium [15]. Ultrasound process is based on the cavitation phenomenon, meaning production, growth, and collapse of bubbles that cause cell rupture, leading the solvent throughout the micro-porous of the damage cell generating internal diffusion and increasing mass transfer. Those bubbles can reach a heating and cooling rate of >1010 K/s, a temperature of 5000 K, approximately, and a pressure about 1000 atm (≈100 MPa) [16].

Using innovative technologies, i.e., ultrasound technology, in combination with green solvents such as vegetable oils and co-solvents for the extraction of carotenoids from fruits and vegetables wastes and/or their by-products, has been in the spotlight for the last several years. The replacement of organic solvents by vegetable oils can find potential applications and advantages as they are safe (non-toxic), natural (plant origin), they can act as a barrier between the carotenoids extracted in the vegetable oil and the atmosphere avoiding oxidation and degradation processes, and they might increase lipid molecules extraction yields due to the lipophilic nature of the vegetable oils [17,18,19,20]. A recent investigation carried out by our research group [21] brings out the ideas above mentioned. We studied the application of HHPAE processes to extract carotenoids from papaya pulp and peel using vegetable oils as extraction solvents. We reported promising results, where lycopene showed great extraction yields (>85% in pulp extracts and >33% in peel extracts) due to the lipophilic nature of the carotenoid. We also detected the need of use a co-solvent, i.e., ethanol, to improve the extraction of the more polar carotenoids, which led us to this study. For the first time, the composition of UAE oil extracts was analyzed in order to identify the carotenoids (xanthophylls, hydrocarbon carotenoids, and carotenoid esters) extracted to the vegetable oil and the healthy potential of the extracts. The findings present an opportunity to revalue and use papaya by-products as a good source of carotenoids for its application in the food, cosmetic, and pharmaceutical sectors.

The objective of this work was to evaluate the extractability of individual carotenoids and carotenoid esters from papaya by-products (pulp and peel) using ultrasound-assisted extraction (UAE) along with green solvents (soybean oil and sunflower oil) in the presence (or absence) of co-solvent (ethanol). The extraction conditions (amplitude, temperature, and extraction time) were optimized using response surface methodology. Furthermore, comparative studies between UAE and conventional extraction were performed in terms of processing procedure and carotenoids content.

## 2. Results and Discussion

### 2.1. Carotenoid UAE from Papaya Pulp and Peel Using Vegetable Oils

The complete characterization of carotenoids and carotenoids esters from papaya cv. Sweet Mary tissues (pulp and peel) using the convectional extraction has been previously reported by Lara-Abia et al. [2], and this information was the guide to identify the carotenoids and carotenoids esters extracted to the vegetable oils. The chromatographic identification (retention time (min), compound identity, absorption maxima (nm), %III/II, %A_b_/A_ii_, [M + H]^+^ (*m/z*), and MS fragmentation pattern (*m/z*) of each lipophilic carotenoid compound found in oil vegetable extracts is described in Table 1. The chromatographic profiles of the papaya pulp and peel extracts obtained from the standard (control) untreated and UAE extracts did not show any differences in carotenoid profiles between using soybean oil and sunflower oil (Figure S1, Supplementary material). In Figure S1, the chromatograms corresponded to the carotenoid profile of UAE extracts obtained at the optimum conditions (high yields) and to the control extracts obtained by the standard protocol for carotenoid analysis [2]. No degradation of carotenoids or carotenoid esters was detected in the UAE extracts, thus carotenoid profiles remained unchanged throughout the extraction process. Seventeen carotenoids were observed in UAE extracts (Table 1). Three of them were free xanthophylls: (9*Z*)-α-cryptoxanthin, (all-*E*)-α-cryptoxanthin, and (all-*E*)-β-cryptoxanthin); ten were xanthophyll esters: (9*Z*)-violaxanthin laurate, (all-*E*)-lutein-3-*O*-myristate, (all-*E*)-antheraxanthin myristate palmitate, (all-*E*)-violaxanthin palmitate, (9*Z*)-neoxanthin dibutyrate, (all-*E*)-β-cryptoxanthin caprate, (all-*E*)-lutein dimyristate, (all-*E*)-β-cryptoxanthin laurate, (all-*E*)-antheraxanthin laurate myristate, and (all-*E*)-β-cryptoxanthin myristate); and finally, four were hydrocarbon carotenoids (all-*E*)-β-carotene, (13*Z*)-lycopene isomer 2, (9*Z*)-lycopene isomer 4, and (all-*E*)-lycopene). In the following section it will be discussed the content of each carotenoid identified in the vegetable oil UAE extracts.

### 2.2. Influence of Independent Variables on the Extraction Yields of Papaya Carotenoids and Carotenoid Esters

A comparison between UAE using soybean oil and sunflower oil as extraction solvents, and different percentages of ethanol as co-solvent, was carried out to study the effect of ultrasounds on the extraction yields of each carotenoids from papaya pulp and peel under different conditions. Great extraction yields of xanthophyll esters compared to hydrocarbon carotenoids and free xanthophylls was observed in all UAE extracts obtained from papaya pulp using soybean oil (Table S1).

The UAE extraction yields of total (*E*)-carotenoids significantly increased (≈16%) when increased ethanol percentage in the green solvent (vegetable oils). This fact can be explained by ultrasounds cavitation process which facilitated fast carotenoid solubilization, while a simultaneous isomerization of some of them could occurred due to the physical conditions that took place when the bubbles collapsed. We detected an increase in β-cryptoxanthin isomers in pulp and peel extracts using soybean oil and sunflower oil after applying ultrasound treatment compared to the control extracts. We also noticed an increase in (*Z*)-lycopene isomers and a decrease in (all-*E*)-lycopene in all UAE extracts, possibly due to the isomerization process that occurred during the treatments. Similar results were obtained by Song et al. [22]; they observed that (all-*E*)-lutein and total trans carotenoids from pumpkin peel increased with the increase in ethanol concentration when the ethanol content was lower than 50% (*v*/*v*). In sunflower oil, UAE pulp extracts the highest yield for xanthophyll esters (Table S2) was obtained with run 11 (yield 40%) which corresponded with a volume of ethanol in vegetable oil of 25%, and an ultrasounds amplitude of 40% for 35 min. However, using soybean oil as solvent the highest total xanthophyll esters extraction yield (48%) was obtained with run 2 (20% amplitude/ 60 min/ 5% ethanol). The extraction of xanthophyll esters with sunflower oil and high ethanol concentration needed less extraction time than using soybean oil to obtain higher carotenoid extraction yields, which could be explained due to the differences in the solvation power of both oils. Sunflower oil mainly contains linoleic acid (poly-unsaturated fatty acid), and soybean oil is composed mainly of oleic acid (mono-unsaturated acid), palmitic acid (saturated fatty acid), and linoleic acid. Thus, sunflower oil is more polar than soybean oil, allowing it to bond more easily to polar carotenoid molecules, i.e., free xanthophylls and xanthophyll esters and, therefore, improve the extraction of total carotenoids from the papaya matrix [21]. Furthermore, using vegetable oils as green solvents turns the extraction of carotenoids into a more effective process because there is no need to eliminate the organic solvents employed in the traditional extraction process. In addition, carotenoid enriched oils can be used directly in the development of food formulations and products, aquaculture feeds, and cosmetic applications. Another advantage of using vegetable oils is the protective role they perform, as they act as a barrier avoiding the oxidation and degradation of the target carotenoids during the extraction [18,23,24]. As seen in the results, the addition of ethanol increased the extraction yield of the mentioned polar carotenoid compounds. Saravana et al. [25] reported higher recoveries of carotenoids and fucoxanthin from brown seaweed (*Saccharina japonica*) using sunflower oil as a co-solvent with supercritical-CO_2_ compared to soybean oil, ethanol, and canola oil. Cardoso et al. [26] observed an increase in β-carotene extraction yield using CO_2_ and 5% of ethanol as the co-solvent. Although several investigations have evaluated the extraction of carotenoids using green solvents, this is the first time a study has combined soybean oil and sunflower oil with ethanol as a co-solvent to improve the extraction of carotenoids and carotenoid esters from papaya tissues.

**Figure 1:**
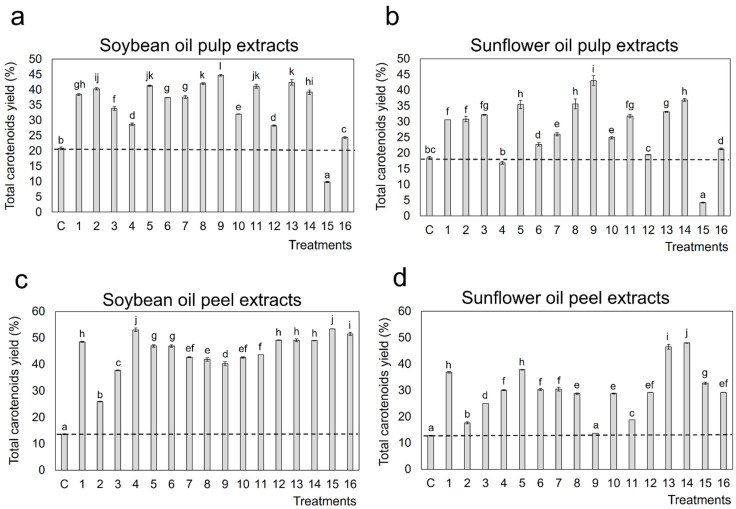
Total carotenoids extraction yield (%) in (**a**) soybean oil and (**b**) sunflower oil UAE pulp extracts and (**c**) soybean oil and (**d**) sunflower oil UAE peel extracts using ethanol as co-solvent. Letter “C” refers to control and the numbers correspond to the combinations of the variables in the CCD listed in Table 2. Superscript letters (a–l) indicate significant differences (*p* < 0.05) between extracts.

Total carotenoids (as the sum of individual carotenoids quantified by HPLC) yields (%) from papaya pulp and peel using soybean oil and sunflower oils are shown in Figure 1. In soybean oil pulp extracts (Figure 1a), the highest total carotenoid extraction yield (45%) was obtained applying UAE processes with 60% amplitude during 10 min and with 20% ethanol (*v*/*v*) (run 9), while the control extract, obtained by standard protocol using no green organic solvents (see Section 3), showed an extraction yield of only 21%. The lowest total carotenoid UAE yield (10%) was obtained with run 15 (20% amplitude/77 min/20% ethanol). Among all the carotenoids identified in UAE soybean oil extracts from papaya pulp, the (all-*E*)-lycopene and their isomers showed the greatest extraction efficiency compared to the control extract (Table S1), due to the non-polar lipophilic nature of the mentioned compounds. Additionally, we observed high papaya xanthophyll esters extraction yields (>24%) in all UAE extracts except for the UAE process conducted at 20%/77 min/ 20% ethanol (run 15) (12% extraction yield), where the extraction yield was lower than the observed for the correspondent control extract (21%). β-carotene was the hydrocarbon carotenoid with lower extraction yields using all UAE process conditions, ranging these values from 3% to 15% (Table S1).

In papaya pulp UAE extracts using sunflower oil (Figure 1b), the highest total carotenoid extraction yield (43%) was obtained with run 9 (60% amplitude/ 10 min/ 20% ethanol) and the lowest total carotenoid extraction yield (4%) was also observed with run 15 (20% amplitude/77 min/20% ethanol), with 10% lower yield than the obtained in the control extract (14%). Among all papaya carotenoids identified in UAE vegetable oil extracts, the highest extraction yields were obtained for β-cryptoxanthin laurate, (13*Z*)-lycopene isomer 2, (9*Z*)-lycopene isomer 4, (21–55%, 13–83%, 13–77%, and 28–95%, respectively) and (all-*E*)-lycopene (Table S2).

On the other hand, Figure 1c shows that the highest total carotenoids extraction yield in soybean oil peel extracts (53%) was observed with run 15 (20% amplitude/77min/20% of ethanol), while the lowest was obtained applying 20% of amplitude during 60 min with 5% of ethanol (*v*/*v*) (run 2). The untreated sample showed 14% of total carotenoids extraction yield. Additionally, it was observed that extracts treated with runs 16, 4, and 12 (40% amplitude/35 min/12.5% ethanol), which correspond to the central points of the CCD, showed high total carotenoids extraction yields (51%, 53%, and 49%, respectively). In Table S3, it is shown the extraction yields of the carotenoids identified in the treated and untreated soybean oil peel extracts. All 17 carotenoid species showed similar extraction yields (≈42%), however (all-*E*)-β-cryptoxanthin laurate (≈73%) and (all-*E*)-lycopene (≈66%) and its isomers (≈50%) showed higher extraction yields than the other lipophilic compounds.

Regarding the sunflower oil peel extracts, higher total carotenoid extraction yields (Figure 1d) were obtained from samples submitted to long extraction times and high amplitude levels. The highest total carotenoid extraction yield (39.3%) was obtained, applying 60% amplitude during 60 min and with 5% ethanol (run 15) (Table S4). As occurred with soybean oil peel extracts, (all-*E*)-β-cryptoxanthin laurate (≈50%) and (all-*E*)-lycopene (≈47%) and its isomers (≈41%) showed higher extraction yields than the other compounds (≈28%). However, the extraction yields in sunflower oil products were lower than when soybean oil was used.

### 2.3. Optimization of UAE of Papaya Carotenoids with Vegetable Oils

The influence of the studied parameters, amplitude (%), time (min,) and co-solvent percentage (% ethanol, *v*/*v*) on the total carotenoid content in the UAE extracts is shown in Table 2. Total carotenoids (measured spectrophotometrically at 450 nm, see Section 3), of UAE extracts from papaya pulp and peel using soybean oil ranged from 12.9 ± 0.7 to 58.7 ± 1.6 and 21.3 ± 0.3 to 52.0 ± 0.9 μg/g vegetable oil, respectively. When sunflower oil was used as solvent, total carotenoids range from 5.6 ± 0.1 to 56.0 ± 1.5 μg/g vegetable oil for papaya pulp and 13.3 ± 0.3 to 39.3 ± 0.5 μg/g vegetable oil for extracts from papaya peel. Table 3, shows the response surface quadratic model and the statistical analysis for the linear, quadratic, and the interactions between the three variables (amplitude (%) (A), time (min) (B), and co-solvent percentage (% ethanol, *v*/*v*) (C)). The *p*-value for the model of UAE extraction from papaya pulp using soybean oil was 0.045 and 0.010 with peel. On the other hand, the *p*-values for the model using sunflower oil and papaya pulp and peel were 0.007 and 0.001, respectively. The obtained *p*-values were less than 0.05, indicating that all models were significant (*p* ≤ 0.05) and could be used to predict and obtain the optimization of carotenoids extraction. Overall, the three independent variables and the quadratic terms brought to bear the significant effects on the total carotenoids extraction within a 95% confidence interval.

The regression equations representing the total carotenoid content (TCC) (μg carotenoids/g vegetable oil) as a function of the independent variables (A) amplitude (%), (B) time (min), and (C) co-solvent (% ethanol *v*/*v*) are:

TCC _(pulp)soybean oil_ = 32.38 + 3.57A + 7.19B + 0.51C + 0.88AB + 1.90AC + 5.40BC + 6.13A^2^ + 0.81B^2^ + 5.05C^2^

TCC _(peel)soybean oil_ = 45.76 + 1.42A + 1.68B + 1.23C + 0.73AB − 5.05AC + 6.07BC − 1.52A^2^ − 3.21B^2^ − 2.16C^2^

TCC _(pulp)sunflower oil_ = 23.00 + 6.02A − 5.84B + 0.41C + 1.65AB + 1.87AC − 9.28BC + 4.53A^2^ + 4.50B^2^ + 4.33C^2^

TCC _(peel)sunflower oil_ = 25.77 + 3.16A + 2.07B − 1.94C + 3.51AB − 4.98AC + 4.89BC + 2.36A^2^ + 0.10B^2^ − 1.43C^2^

The significance of the model is given by the value of determination coefficient r^2^ and adjusted r^2^, which should be close to the determination coefficient r^2^ for a good statistical model. In all four models, both the determination coefficient r^2^ and adjusted r^2^ are >0.90 and with very similar values, which indicates a great accordance between the experimental data and the theoretical data. Additionally, none of the mathematical models showed non-significant lack-of-fit (*p* > 0.05) indicating that the equations for each model are appropriated to use in the optimization process (Table 3). Overall, this analysis showed a better fit for peel extracts compared to pulp extracts. Nonetheless, all models were valid to continue with the optimization process, and therefore the models represented by the regression equations can be confidently used to handle the design.

**Figure 2:**
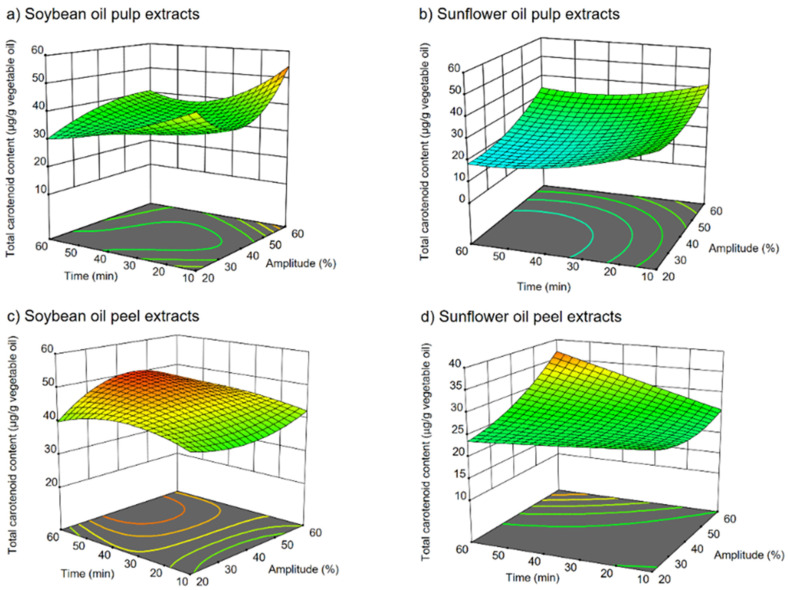
Response surface methodology for the extraction of total carotenoids (μg carotenoids/g vegetable oil) from papaya pulp using (**a**) soybean oil and (**b**) sunflower oil; and from papaya peel using (**c**) soybean oil and (**d**) sunflower oil.

Response surface plots obtained for total carotenoid content as a function of amplitude, time, and volume of co-solvent (Figure 2) showed the influence of the independent variables on the extraction of total carotenoids from papaya pulp using soybean oil (Figure 2a) and sunflower oil (Figure 2b), and from papaya peel using soybean oil (Figure 2c) and sunflower oil (Figure 2d). In all the plots, the independent variable ethanol was set as the actual factor with a value of 12.5% to better understand the influence of the amplitude and the extraction time. Figure 2a,b show that the total carotenoids content increased with high amplitude values and higher to moderate extraction times, which agrees with the observed data in Table 3, where the highest carotenoid contents in pulp UAE extracts were and 58.7 ± 1.6 and 56.0 ± 1.5 μg/g oil using soybean and sunflower oil, respectively, and using a 60% amplitude, 60 min, and 20% of ethanol (run 9); in contrast, the lowest carotenoid contents using soybean (12.9 ± 0.7 μg/g oil) and sunflower oil and (5.06 ± 0.1 μg/g oil) were obtained with treatments at 20% amplitude, 77 min, and 20% of ethanol (run 15). Other authors, such as Ordonez-Santos et al. [27], obtained similar results for UAE extraction of peach palm peel. They observed an increase in the extraction of carotenoids from peach palm peel using sunflower oil as alternative solvent when increasing the amplitude, time, and temperature. The increase in extraction yields with high amplitude values may be due the appearance of greater number of cavitation micro-bubbles, facilitating the collapse of the bubbles more drastically as the ultrasonic intensity increases, which led to the disruption of the tissue cell walls accelerating the diffusion of carotenoids into the solvent. Several authors [18,28,29] have reported that an increase in the extraction temperature up to 40 °C increases the extraction of carotenoids. This may be because it favors the mass transfer rate as with higher temperature the solubility of the carotenoids increased, possibly due to the breaking up of carotenoid complexes with proteins and the reduction in the solvent viscosity. However, Dong et al. [28] reported that temperatures higher than 40 °C may also dissolve impurities and some thermal labile components may decompose. For this reason, in the present study, temperatures above 30 °C were not used in any case.

On the other hand, according to the tendency observed in the response surface plot for papaya peel soybean oil UAE extracts (Figure 2c), the highest total carotenoid content could be achieved with higher extraction times and low to moderate amplitude values, which concurs with the obtained data in Table 2. Total carotenoid content of 52.0 ± 0.9 μg/g oil was achieved applying 20% amplitude for 77 min with 20% ethanol (run 15), which is the same run for which papaya pulp with soybean and sunflower oil UAE extracts showed their lowest total carotenoid content, as mentioned before. Run 2 (20% amplitude/60 min/5% ethanol) extracted the lowest total carotenoid content (21.3 ± 0.3 μg/g oil) from papaya peel soybean extracts. In Figure 2d, we observed an increase in total carotenoids content when high amplitude levels and higher extraction times were applied, which corresponds to the highest total carotenoid content (39.3 ± 0.5 μg/g oil) observed in UAE sunflower oil papaya peel extracts obtained at 60% amplitude for 60 min and with 5% ethanol (run 14). The lowest total carotenoid content (13.3 ± 0.3 μg/g oil) was obtained with run 9 (60% amplitude/ 10 min/ 20% ethanol). The control extracts (obtained by standard extraction protocol without ultrasounds) in papaya pulp extracts using soybean (19.5 ± 0.6 μg/g oil) and sunflower oil (20.1 ± 0.7 μg/g oil) were lower than the carotenoid content observed in the UAE extracts, except for the papaya pulp UAE extracts obtained by a process using 20% amplitude/ 77 min/ 20% ethanol (run 15), possibly due to carotenoid losses associated with the longer UAE extraction time, producing degradation of these compounds. Differences between papaya pulp and peel UAE extracts applying the same UAE extraction conditions could be due to their different carotenoid content among both vegetable tissues. Papaya peel is formed by collenchyma cells, which are thicker, and with more pectin than pulp cells, which are thinner [2]; thus, the optimal conditions to extract carotenoids from papaya peel were those with a long extraction time and low ultrasonic intensity.

A positive correlation using the regression coefficient (r^2^) was observed between total carotenoids content (μg carotenoids/g oil) and color determination (∆E) (Figure S2). In soybean and sunflower oil pulp extracts, the regression coefficients were 0.96 and 0.90, respectively, and r^2^ of 0.95 and 0.91 were obtained for soybean oil and sunflower oil peel extracts, respectively. The results indicated that the more colored vegetable oil extracts contained higher carotenoid content after applying UAE than those which showed less color intensity, including untreated samples. Similar results were obtained by Nowacka and Wedzik [30] who observed better color retention in carrot samples treated with ultrasounds compared to untreated samples. As mentioned before, the combination of low extraction times and low amplitude values may not cause enough significant changes in the microstructure of the vegetable cell that allows the extraction of carotenoids from the matrix and, therefore, increase the color of the vegetable oil (extracting solvent).

## 3. Materials and Methods

### 3.1. Plant Material

Mature fruits of red-fleshed papaya (*Carica papaya*, cv. Sweet Mary) were collected from greenhouses cultivars in Güimar (Santa Cruz de Tenerife, Canary Islands, Spain). The fruits were selected according to their size, shape, percentage of external coloration, and lack of visual defects. Maturity level 5 was chosen for all papaya fruits according to the ripening stages reported by Ramos-Parra et al. [31]. Physical and physicochemical characteristics of papaya fruits (Table S5) were evaluated as described before by Plaza et al. [32].

Papaya fruits were washed, peeled, and sliced and seeds were removed. Pulp and peel tissues were separated and cut manually. Portions of papaya tissues (approximately 150 g) were placed in plastic bags, vacuum sealed, frozen in liquid nitrogen, and freeze-dried for 5 days at −45 °C and 1.3 × 10^−3^ MPa (LyoBeta 15, Azbil Telstar SL, Terrasa, Spain). The freeze-dried materials were ground to a fine particle size (<2 mm) (Grindomix GM200, Retsch, Germany), vacuum packed in plastic bags and stored at −20 °C until used in experiments.

### 3.2. Standards and Chemicals

Standards for (all-*E*)-zeaxanthin (HPLC 97%, synth., cryst.), (all-*E*)-neoxanthin (HPLC 97%, isolated, cryst.), (all-*E*)-violaxanthin (HPLC 95%, isolated, cryst.), (all-*E*)-β-carotene (HPLC 96%, synth., cryst.), (all-*E*)-α-carotene (HPLC 97%, synth., cryst.), and (all-*E*)-β-cryptoxanthin (HPLC 97%, synth., cryst.) were purchased from CaroteNature (Ostermundigen, Switzerland). Lutein (X6250 from marigold), lycopene (L9879, ≥90%, from tomato), and (all-*E*)-β-apo-8′-carotenal (10,810, ≥96%, (UV)) were purchased from Sigma-Aldrich (St. Louis, MO, USA).

Milli-Q water was obtained from a Millipark^®^ Express 40 system (Merk-Millipore, Darmstadt, Germany); methyl tert-butyl ether (MTBE), methanol (MeOH), diethyl ether, tetrahydrofuran (THF), ethanol absolute, and acetone were purchased from VWR International (Radnor, PA, USA); magnesium carbonate (MgCO_3_) and butylated hydroxytoluene (BHT) were obtained from Acros Organics (Geel, Belgium); sodium chloride (NaCl), potassium hydroxide (KOH), and anhydrous sodium sulphate (Na_2_SO_4_) were purchased from Panreac Quimica (Barcelona, Spain). Soybean (La Española Soy Plus^®^) and sunflower (Aliada^®^) refined oils and were purchased from a local market in Madrid (Spain).

### 3.3. Ultrasound-Assisted Extraction of Papaya Carotenoids

The preparation of the samples was carried out mixing freeze-dried papaya tissues with each vegetable oil, keeping a solid:solvent ratio of 2:10 (g/mL). The ratio was selected according to the results reported by Goula et al. [18]. Ethanol (>99.5% *v*/*v*) was used as the co-solvent, in a range of 5–20% (*v*/*v*), to improve the extraction of the more polar carotenoids. The mixture (adding co-solvent when required) was homogenized with a vortex for 1 min. All UAE treatments associated with each run are indicated in Table 2. Afterwards, papaya preparations were treated using an ultrasonic processor operating at 400 W of maximum nominal output power, 20 kHz of frequency (Digital Sonifier; Branson Ultrasonics Corporation, Danbury, CT, USA), 20–60% of amplitude, and pulsed operating mode (2 s on/1 s off). The amplitude control of the sonicator allowed the vibrations in the probe to be set at any desired level in the 10–100% range of nominal power. A microtip horn of 13 mm diameter (Branson Ultrasonics Corporation, Danbury, CT, USA) was immersed inside the sample in a depth of 2 cm regarding the liquid surface contained in the beaker glass with 15 mL of papaya preparations. The temperature was constantly controlled with an external water bath to avoid temperatures over 30 °C. Soybean and sunflower commercial oils were used as alternative solvents. The amplitude level range was 20–60%, the extraction time 10–60 min, and the percentage of the co-solvent 5–20% (ethanol). All treatments were made in triplicate. After the extraction process, samples were centrifuged at 15,000× *g* for 10 min at 4 °C. The pellet was discarded, and the carotenoid-enriched oils were collected. All carotenoid-rich oils were stored at −80 °C until carotenoid analysis.

Carotenoid content in vegetable oils was expressed as absolute content (μg carotenoid/g vegetable oil) or % carotenoid extraction yield ((μg carotenoids in the oil extract/g of freeze-dried tissue)/(μg carotenoids/g of freeze-dried tissue) × 100).

### 3.4. Experimental Design

A central composite design (CCD) was set up to evaluate the effect of time, amplitude, and vegetable oil/ethanol solvent (independent variables) on the total carotenoid content (dependent variable). A total of sixteen different combinations, including three replicates of center point were obtained with the composite design (Table 2). The selected optimization parameter was carotenoid content, expressed as μg carotenoids/g vegetable oil. The effects were studied at two experimental levels −1 and +1. Each combination was performed in triplicate. The data obtained were modelled with a second-order polynomial equation (Equation (1)).

(1) $$Y=\beta_{0}+\sum_{i=1}^{k}\beta_{i}X_{i}+\sum_{i=1}^{k}\beta_{\mathrm{ii}}X_{i}^{2}+\sum_{i>j}^{k}\;\beta_{\mathrm{ij}}X_{i}X_{J}$$

where Y is the dependent variable to be evaluated, Xi and Xj are the independent factors, β0 is the intercept, βi corresponds to the linear coefficients, βii indicate the quadratic coefficients, βij corresponds to the cross-product coefficients, and k would be the total number of independent factors. The central composite design and the obtained data were carried out using the Design-Expert 11.1.2.0 (Stat-Ease Inc., Minneapolis, MN, USA) software package.

### 3.5. HPLC Analysis of Carotenoids in Vegetable Extracts and Papaya Tissues

#### 3.5.1. Carotenoid Extraction from Ultrasound Vegetable Oils Extract

The extraction of carotenoids from soybean and sunflower oil US treated samples and untreated (control) samples was performed following the methodology proposed by Mutsokoti et al. [33] with some modifications. First, 0.5 g of carotenoid enriched oil was mixed with 10 mL of a solvent mixture (50:25:25, *v*/*v*/*v*, hexane/acetone/ethanol) and 0.1 g NaCl. Samples were stirred for 20 min at 4 °C in darkness and an absence of oxygen. Then, 3 mL of reagent grade water was added and mixed during 10 min at 4 °C in the same conditions. Samples were placed in separation funnels and the carotenoid-rich organic phase was collected in amber flasks, which was evaporated on a rotatory evaporator with controlled temperature (20 °C) and dissolved in 2 mL of hexane. The obtained extract was first used to measure spectrophotometrically total carotenoids content. Then, it was vacuum dried in a rotatory evaporator at 20 °C, dissolved to 2 mL with MeOH/MTBE/H_2_O (45.5:52.5:2, *v*/*v*/*v*), and analyzed by HPLC. The commercial soybean oil and sunflower oil carotenoid content was also analyzed by HPLC-DAD (data not shown), concluding that no carotenoids were found in either of the two oils that could interfere with the carotenoids extracted from the freeze-dried papaya tissues.

#### 3.5.2. Carotenoid Extraction from Control Vegetable Oils Extracts

In order to elucidate the advantages of US application on carotenoids and carotenoid esters extraction from papaya fruit tissues, an extraction of carotenoids from papaya tissues was performed mixing freeze-dried papaya pulp and peel with soybean oil and sunflower oil in a ratio of 2:10 g/mL by homogenization. The mixture was homogenized at 25,000 rpm for 2 min using Ultra-turrax homogenizer (T-25 Digital, IKA work Inc., Breisgau, Germany) with an external sample cooling to avoid temperature increase. Then, it was centrifuged at 15,000× *g* for 10 min at 4 °C, and the supernatant was collected. The collected oil extracts (named control extracts) were stored at −80 °C until carotenoid analysis. Control oil extracts were analyzed as described above.

#### 3.5.3. Extraction of Carotenoids from Papaya Tissues

Conventional extraction of carotenoids and their esters from the freeze-dried papaya tissues (pulp and peel) was performed according to the procedure reported by Cano et al. [34] with some modifications. First, 1 g of the freeze-dried sample was mixed with 0.5 g magnesium carbonate and 50 μL of (all-*E*)-β-apo-8’-carotenal (0.40 mg/mL), as internal standard. Then, 20 mL of tetrahydrofuran (THF) stabilized with 0.1% (*w*/*v*) of butylated hydroxytoluene (BHT) were added for the extraction. The sample was homogenized in an Omnimixer (OMNI Macro S^®^, OMNI International, Kennesaw, GA, USA) at 3000 g for 3 min and placed in an ultrasonic water bath (3000514 model, 50/60 Hz, 360 W, J.P. Selecta S.A., Barcelona, Spain) for 30 min. The extract was centrifuged at 15,000× *g* for 10 min at 4 °C and the supernatant was collected. Subsequently, 20 mL of acetone was added to the pellet and the sample was extracted again. Re-extraction of the recovered solids was carried out 3 times until a colorless residue was obtained. Supernatants were combined in the same separation funnel, where 20 mL of diethyl ether was added; when an emulsion was formed, 20 mL of saturated water with 30% (*w*/*v*) NaCl was added. The organic phase was collected and dried with 2.5 g of anhydrous sodium sulphate over 10 min at room temperature. The sample was filtered through Whatman No. 1 filter paper and the filtrated obtained was transfer to a round amber flask and vacuum dried in a rotatory evaporator at 30 °C. Finally, the extract was dissolved to 2 mL with MeOH/MTBE/H_2_O (45.5:52.5:2, *v*/*v*/*v*) and filtered through a 0.45 μm filter to be analyzed immediately by HPLC. All procedures were carried out at room temperature and in dim light to avoid carotenoid isomerization and degradation.

### 3.6. Carotenoid Analysis by HPLC-DAD

Carotenoids in papaya freeze-dried tissues and in carotenoid-rich oils obtained by UAE were analyzed following the methodology reported by Cano et al. [34]. Carotenoids and carotenoids esters identification and quantification were performed in an Agilent 1200 HPLC System (Agilent Technologies, Santa Clara, CA, USA) with a diode array detector, using a reverse-phase C_30_ column (YMC-Pack YMC C30, 250 × 4.6 mm i.d., S-5μm, YMC Co., Ltd., Kyoto, Japan). The solvents used were a gradient of methanol/MTBE/water (81:14:4, *v*/*v*/*v*, eluent A) and methanol/MTBE (10:90, *v*/*v*, eluent B), both containing 1 g/L of ammonium acetate. The elution gradient was linear, starting at 100% A and ending with 100% B, in 60 min. The injection volume was 40 μL, the operation temperature was set at 32 °C and the flow rate was set at 1 mL/min. Free and esterified carotenoids were monitored with the UV–Vis photodiode array detector set at 450 nm and additional UV–Vis spectra were recorded in the range of 200–600 nm.

Carotenoid identification in papaya extracts was carried out by using retention times, UV–Vis absorption spectra (λmax, spectral fine structure (%III/II), peak cis intensity) and by comparing chromatographic and spectrophotometric properties with commercial standards as previously was described by Lara-Abia et al. [2]. Additionally, data available in the literature was used to compare mass spectrum of unidentified compounds when standards were unavailable [34,35,36,37]. (all-*E*)-lycopene, (all-*E*)-β-carotene, (all-*E*)-α-carotene, (all-*E*)-β-cryptoxanthin, (all-*E*)-lutein, (all-*E*)-zeaxanthin, (all-*E*)-violaxanthin, (all-*E*)-neoxanthin, and trans-β-apo-8′-carotenal were quantitated using their respective calibration curves, preparing different concentrations (0.1–200 μg/mL) for each standard (Figure S3). Carotenoid esters were quantified with the corresponding free carotenoid calibration. Vitamin A value was calculated as retinol activity equivalent (RAE) per 100 g of fresh weight, following the equation RAE = (μg of β-carotene/12) + (μg of other pro-vitamin A carotenoids, such as β-cryptoxanthin and β-cryptoxanthin esters)/24) Institute of Medicine (US) [38]. Results were expressed as micrograms of the corresponding carotenoid per 100 g of fresh weight.

### 3.7. Carotenoid Analysis by Liquid Chromatography-Mass Spectrometry (LC-MS/MS (APCI^+^))

LC/MS analyses were performed with the same HPLC system described previously in order to complete the identification of individual carotenoids present in carotenoid-rich oil samples. According to Breithaupt and Schwack [39], the HPLC system was coupled on-line to an Agilent mass spectrometry detector with APCI source model G1947B compatible with the LCMS SQ 6129 equipment, according to positive ion mass spectra of the column eluted of 13,000 Th/s (peak width 0.6 Th, FWHM). The nebulizer temperature was 350 °C and potential of +2779 kV was used on the capillary. Nitrogen was used as the drying gas at flow rate of 60 L/min and as nebulizing gas at pressure of 50 psi. Corona was set at 4000 Na in positive ion mode, and the devaporizer temperature was set at 400 °C. The collision gas was helium and the fragmentation amplitude was 0.8–1.2 V. Chromatographic conditions were the same as described for the quantitative analysis of carotenoids.

### 3.8. Determination of Total Carotenoid Content of Carotenoid- Oils Extracts

Total carotenoid content was determined spectrophotometrically at 450 nm using a UV–Vis spectrophotometer (Specord 210 Plus, Analytik Jena). After carotenoids extraction procedure, the volume of the carotenoid oils extracts was noted, and the absorbance was measured against absolute hexane used as blank. Hexane solutions were analyzed under diminished light. Total carotenoid content (μg carotenoids/g vegetable oil) was calculated according to Equation (2).

(2) $$\mathrm{Total}\;\mathrm{carotenoid}\;\mathrm{content}\;(\mu g/g)=[(A\;\times \;V)/(E_{1\mathrm{cm}}^{1\%}\times \;P\;\times 100)]\;\times \;10^{6}$$

where, A is absorbance at 450 nm, V is total volume of dissolution (mL), $E_{1\mathrm{cm}}^{1\%}$ is extinction coefficient of mixture of carotenoids in hexane (ξ = 2500 dL/g cm, according to Melendez-Martinez et al. [40]), and P is oil extract weight (g).

### 3.9. Color Determination of Carotenoid-Rich Vegetable Oils

Colorimetric determinations (CIELab system) of carotenoid oil extracts samples were performed according the procedure described by Schweiggert et al. [41]. Extracts were analyzed using a Konica Minolta CM-3500d equipment (Madrid, Spain), applying D65 light mode and 10° observer angle. Extracts were exposed to light within a wavelength range of 380–780 nm. Total color differences were determined in each extract against the particular solvent (soybean or sunflower refined oil) used as a blank. Total color differences (∆E) were calculated with the means of the colorimetric values ∆L* (brightness), ∆a* (red–green proportion) and ∆b* (yellow–blue proportion). Color determinations of the oil samples were performed three-fold. The total color difference for each extract was calculated using Equation (3):

(3) $$\Delta E^{*}={\left(\Delta {L^{*}}^{2}+\Delta {a^{*}}^{2}+\Delta {b^{*}}^{2}\right)}^{1/2}$$

### 3.10. Statistical Analysis

All analyses were performed in triplicate and the results presented as means ± standard deviation. One-way analysis of variance (ANOVA) and Tukey-b test were used to evaluate the significance of the differences between pairs of means values applying *p* ≤ 0.05 of significance level. SPSS software (version 20.0, SPSS Statistical Software, Inc., Chicago, IL, USA) was used to perform the statistical analyses of the data obtained from the experiments conducted to evaluate the effect of ultrasound-assisted extraction on carotenoid extraction yield using vegetable oils. The same statistical software was used to analyze the data obtained from total carotenoids content and color analyses.

## 4. Conclusions

The effects of ultrasound-assisted extraction (UAE) conditions on extraction yields of total and individual carotenoids from papaya pulp and peel were studied using central composite design (CCD) and response surface methodology (RSM). Three extraction factors amplitude (%), time (min), and volume of ethanol (%) were evaluated. Soybean oil and sunflower oil were used as green extraction solvents and ethanol as a co-solvent. As a result of this investigation, it was observed that short extraction time, high amplitude level, and high ethanol percentage were suitable to extract carotenoids from papaya pulp extracts using either soybean oil (59 ± 2 μg carotenoids/g oil) or sunflower oil (56 ± 2 μg carotenoids/g oil), but the opposite occurred when extracting carotenoids from papaya peel (52 ± 1 μg carotenoids/g soybean oil and 39 ± 1 μg carotenoids/g sunflower oil). The beneficial role of using vegetable oils to extract carotenoids from papaya tissues has been demonstrated, as it improved the solvation power (high extraction yields) and prevented carotenoids degradation during the extraction process. In the UAE process applied to papaya tissues, higher extraction yields of hydrocarbon carotenoids ((all-*E*)-lycopene and its isomers) compared to free xanthophylls and xanthophyll esters were observed. Additionally, the application of ultrasounds to extract the papaya carotenoids promoted the isomerization of some of its carotenoids such as (all-*E*)-β-cryptoxanthin and (all-*E*)-lycopene. The addition of ethanol as co-solvent to the vegetable oils had a positive effect for the extraction of most polar carotenoids, specifically xanthophyll esters. The application of vegetable oils as alternative green solvents for the UAE extraction of carotenoids from plant materials is an efficient alternative to obtain healthy carotenoid-based ingredients and nutraceuticals.

## Figures and Tables

**Table 1:** Chromatographic identification ^1^ of carotenoids and carotenoid esters obtained from direct (unsaponified) pulp and peel papaya (*Carica papaya* L. cv. Sweet Mary) UAE extracts using vegetable oils (soybean and sunflower oil) and ethanol as green extraction solvents.

**Peak**	**Rt (min)**	**Compound Identity**	**HPLC-DAD UV/Vis Absorption Maxima (nm)**	**%III/II**	**%A_b_/A_II_**	**[M + H]^+^ *m/z***	**HPLC/APCI^+^ MS Fragmentation Pattern (*m/z)***
1	13.4	(9*Z*)-α-cryptoxanthin	412, 437, 466	0	0	n.d.	n.d.
2	13.9	(all-*E*)-α-cryptoxanthin	(413), 435, 464	61	n.c.	553	535 [M + H − 18]^+^, 479, 461 [M + H − 92]^+^, 439
3	16.0	(all-*E*)-β-cryptoxanthin	(426), 450, 476	18	n.c.	553	535 [M + H − H_2_O ]^+^, 461 [M + H − 92]^+^
4	23.7	(9*Z*)-violaxanthin laurate	388, 413, 436, 466	92	0	783	765 [M + H − H_2_O]^+^, [M + H − 18]^+^, 747 [M + H − 2H_2_O]^+^, [M + H − 18 − H_2_O]^+^, 565 [M + H − 12:0 − H_2_O]^+^, [M + H − 12:0 − 18]^+^
5	24.0	(all-*E*)-lutein-3-*O*-myristate	401, 426, 472	0	0	n.d.	533 [M + H − 228 − 18]^+^, 495 [M + H − 228 − 56]^+^, 459 [M + H − 228 − 92]^+^, 429, 441
6	24.3	(all-*E*)-β-carotene	(428), 450, 476	16	0	537	457 [M + H − 80]^+^, 445 [M + H − 92]^+^, 400 [M + H − 137]^+^, 269 [M + H − 268]^+^, 177 [M + H − 360]^+^, 137 [M + H − 400]^+^
7	25.7	(all-*E*)-antheraxanthin myristate palmitate	421, 443, 467	31	0	1033	1015 [M + H − 18]^+^, 941 [M + H − 92]^+^, 805 [M + H − 14:0]^+^, 787 [M + H − 14:0 − 18]^+^, 771 [M + H − 16:0]^+^,759 [M + H − 16:0 − 18]^+^, 549 [M + H − 14:0 − 16:0]^+^, 531 [M + H − 14:0 − 16:0 − 18:0]^+^
8	26.1	(all-*E*)-violaxanthin palmitate	416, 441, 469	nc	nc	839	821 [M + H − 18]^+^, 803 [M + H − 18 − 18]^+^, 747 [M + H − 16:0]^+^, 729 [M + H − 92 − 18]^+^, 583 [M + H − 256]^+^, 565 [M + H − 18 − 16:0]^+^, 547 [M + H − 16:0 − 18 − 18]^+^
9	27.1	(9*Z*)-neoxanthin dibutyrate	327, 412, 436, 464	80	16	741	723 [M + H − 18]^+^, 653 [M + H − 4:0]^+^, 649 [M + H − 92]^+^, 635 [M + H − 4:0 − 18]^+^, 631 [M + H − 92 − 18]^+^, 565 [M + H − 4:0 − 4:0]^+^, 547 [M + H − 4:0 − 4:0 − 18]^+^
10	27.5	(all-*E*)-β-cryptoxanthin caprate	428, 450, 476	nc	n.c.	707	615 [M + H − 27]^+^, 535 [M + H − 100]^+^, 443 [M + H − 11]^+^, 442 [M + H − 16]^+^
11	28.6	(all-*E*)-lutein dimyristate	422, 446, 474	38	0	n.d.	761 M + H − 14:0]^+^, 669 [M + H − 92]^+^, 553 [M + H − 14:14:0]^+^
12	29.3	(all-*E*)-β-cryptoxanthin laurate	421, 451, 478	25	0	735	643 [M + H − 92]^+^, 535 [M + H − 12:0]^+^, 479 [M + H − 56 − 12:0]^+^, 443 [M + H − 92 − 12:0]^+^
13	31.2	(all-*E*)-antheraxanthin laurate myristate	418, 442, 470	33	0	977	959 [M + H − 18]^+^, 777 [M + H − 12:0]^+^, 749 [M + H − 14:0]^+^, 759 [M + H − 12:0 − 18]^+^, 731 [M + H − 14:0 − 18]^+^, 549 [M + H − 12:0 − 14:0]^+^, 531 [M + H − 12:0 − 14:0 − 18]^+^
14	32.0	(all-*E*)-β-cryptoxanthin myristate	424, 448, 476	9	0	763	671 [M + H − 92]^+^, 535 [M + H − 14:0]^+^, 443 [M + H − 14:0 − 92]^+^
15	32.9	(13*Z*)-lycopene isomer 2	442, 465, 493	0	0	537	481 [M + H − 42]^+^, 467 [M + H − 35]^+^, 455 [M + H − 100]^+^, 427 [M + H − 61]^+^, 413 [M + H − 88]^+^, 399 [M + H − 24]^+^, 387 [M + H − 42]^+^
16	36.9	(9*Z*)-lycopene isomer 4	440, 465, 496	0	0	537	481 [M + H − 11]^+^, 467 [M + H − 32]^+^, 455 [M + H − 79]^+^, 427 [M + H − 48]^+^, 413 [M + H − 30]^+^, 399 [M + H − 42]^+^, 387 [M + H − 38]^+^
17	41.7	(all-*E*)-lycopene	418, 443, 471, 502	6	0	537	457 [M + H − 80]^+^, 413 [M + H − 124]^+^, 177 [M + H − 360]^+^, 137 [M + H − 400]^+^, 121 [M + H − 416]^+^

Rt.: retention time. n.c.: %III/II was not calculated due to the poor definition of the UV/Vis spectrum or because it was not detected. n.d.: [M + H]^+^ or MS/MS fragments were not detected. ^1^ The complete characterization of carotenoids and carotenoid esters of papaya cv. Sweet Mary was based on information reported in previous studies [2].

**Table 2:** Combination of the independent variables obtained with CCD and the obtained total carotenoid content in papaya (*Carica papaya* L. cv. Sweet Mary) pulp and peel extracts measured spectrophotometrically at 450 nm.

Run ^1^	Amplitude (%)	Time (min)	EtOH (%)	Carotenoid Content (μg/g Vegetable Oil) ^2^			
				Pulp		Peel	
				Soybean Oil	Sunflower Oil	Soybean Oil	Sunflower Oil
1	60	60	20	44.2 ± 1.8 ^b^	33.8 ± 1.0 ^a^	42.0 ± 1.1 ^b^	33.6 ± 0.7 ^a^
2	20	60	5	46.7 ± 0.7 ^d^	36.9 ± 1.1 ^c^	21.3 ± 0.3 ^b^	14.5 ± 0.3 ^a^
3	40	1	12.5	40.6 ± 1.0 ^c^	39.9 ± 1.4 ^c^	33.4 ± 1.0 ^b^	21.8 ± 0.6 ^a^
4	40	35	12.5	33.9 ± 1.0 ^c^	18.2 ± 0.5 ^a^	47.2 ± 0.7 ^d^	26.8 ± 0.4 ^b^
5	74	35	12.5	50.3 ± 1.7 ^c^	42.7 ± 0.8 ^b^	42.0 ± 0.9 ^b^	35.8 ± 0.6 ^a^
6	10	35	12.5	41.5 ± 1.1 ^b^	26.7 ± 0.5 ^a^	41.0 ± 0.8 ^b^	27.4 ± 0.7 ^a^
7	20	10	5	43.1 ± 1.0 ^b^	28.6 ± 0.3 ^a^	40.1 ± 0.7 ^b^	27.8 ± 0.4 ^a^
8	20	10	20	54.9 ± 2.2 ^d^	44.4 ± 0.9 ^c^	34.9 ± 0.7 ^b^	22.2 ± 0.2 ^a^
9	60	10	20	58.7 ± 1.6 ^c^	56.0 ± 1.5 ^c^	33.6 ± 0.6 ^b^	13.3 ± 0.3 ^a^
10	40	35	1	34.4 ± 1.1 ^c^	27.2 ± 0.5 ^b^	39.1 ± 1.3 ^c^	22.6 ± 0.7 ^a^
11	40	35	25	49.4 ± 1.2 ^c^	38.5 ± 0.8 ^b^	40.3 ± 1.1 ^b^	19.5 ± 0.2 ^a^
12	40	35	12.5	33.8 ± 0.7 ^b^	23.4 ± 0.7 ^a^	45.8 ± 1.4 ^c^	24.9 ± 0.6 ^a^
13	60	10	5	57.4 ± 1.6 ^b^	40.6 ± 0.7 ^a^	45.5 ± 1.1 ^a^	38.2 ± 1.5 ^a^
14	60	60	5	44.6 ± 1.5 ^ab^	46.5 ± 1.4 ^b^	43.9 ± 0.8 ^ab^	39.3 ± 0.5 ^a^
15	20	77	20	12.9 ± 0.7 ^b^	5.6 ± 0.1 ^a^	52.0 ± 0.9 ^d^	31.9 ± 0.6 ^c^
16	40	35	12.5	29.2 ± 1.1 ^a^	25.6 ± 0.6 ^a^	47.2 ± 1.4 ^b^	26.0 ± 0.3 ^a^
C	-	-	-	19.5 ± 0.6 ^b^	20.1 ± 0.7 ^b^	10.1 ± 0.2 ^a^	9.4 ± 0.2 ^a^
Variables		Factors		Levels			
				−1		1	
Amplitude (%)		A		20		60	
Time (min)		B		10		60	
Ethanol in Solvent (%)		C		5		20	

^1^ Letter “C” refers to control, and the numbers correspond to the combinations of the variables in the CCD. ^2^ All results are specified as the mean of two independent determinations ± standard deviation. Results with different alphabets in the same row are significantly different (*p* < 0.05) from each other.

**Table 3:** Analysis of variance and model regression coefficient of independent variables (amplitude (%), time (min), and ethanol volume (%) in solvent (*v*/*v*)).

Statistical Data ^1^	Soybean Oil						Sunflower Oil					
	Pulp Extracts			Peel Extracts			Pulp Extracts			Peel Extracts		
	Regression Coefficient	F-Value	*p*-Value	Regression Coefficient	F-Value	*p*-Value	Regression Coefficient	F-Value	*p*-Value	Regression Coefficient	F-Value	*p*-Value
Model	32.38	21.65	0.045	45.76	93.04	0.011	23.00	9.13	0.007	25.77	20.57	0.001
A-Amplitude	3.57	1.98	0.295	1.42	3.96	0.185	6.02	17.42	0.006	3.16	26.61	0.002
B-Time	−7.19	0.11	0.769	1.68	58.92	0.017	−5.84	13.87	0.010	2.07	9.69	0.021
C-EtOH	0.51	12.32	0.073	1.23	4.25	0.175	0.41	0.08	0.785	−1.94	10.21	0.019
AB	0.88	0.17	0.723	0.73	10.98	0.080	1.65	0.90	0.380	3.51	22.43	0.003
AC	1.90	3.52	0.202	−5.05	283.13	0.004	1.87	1.03	0.350	−4.98	40.45	0.001
BC	−5.40	26.79	0.035	6.07	420.01	0.002	−9.28	28.25	0.002	4.89	43.64	0.001
A^2^	6.13	29.48	0.032	−1.52	50.79	0.019	4.53	7.50	0.034	2.36	11.32	0.015
B^2^	0.81	16.22	0.057	−3.21	46.56	0.021	4.50	5.76	0.053	0.10	0.02	0.904
C^2^	5.05	14.34	0.063	−2.16	91.41	0.011	4.33	6.81	0.040	−1.43	4.16	0.088
Lack of fit		13.21	0.072		9.41	0.092		2.20	0.336		7.22	0.125
R^2^	0.993			0.998			0.932			0.969		
Adjusted R^2^	0.947			0.988			0.830			0.922		
Adequate precision	18.235			40.604			12.210			14.847		

^1^*p* ≤ 0.05 indicate model terms are significant.

## Data Availability

Not applicable.

## Supplementary Materials

The following supporting information can be downloaded. Figure S1: C_30_ reversed-phase chromatograms of carotenoids and carotenoid esters identified from papaya (*Carica papaya* L. cv. Sweet Mary) in soybean oil pulp (a) control and (b) run 9 (60%/10 min/20% EtOH) extracts, and in peel (c) control and (d) run 14 (60%/ 60 min/ 5% EtOH) extracts; also, in sunflower oil pulp (e) control and (f) run 9 extracts, and in peel (g) control and (h) run 14 extracts. Peak identities in Table 1, Figure S2: Correlation analysis using the regression coefficient (r^2^) between total carotenoids content (μg carotenoids/g vegetable oil) and color determination (∆E) in (a) soybean oil and (b) sunflower oil pulp extracts, and (c) soybean oil and d) sunflower oil peel extracts. Letter “C” refers to control and treated samples correspond to the combinations of the variables in the CCD listed in Table 2, Figure S3: Calibration curves of carotenoid standards. Table S1: Carotenoid extraction yields^1^ obtained from papaya (*Carica papaya* L. cv. Sweet Mary) pulp tissue applying UAE using soybean oil and ethanol as green extraction solvent, Table S2: Carotenoid extraction yields^1^ obtained from papaya (*Carica papaya* L. cv. Sweet Mary) pulp tissue applying UAE using sunflower oil and ethanol as green extraction solvent, Table S3: Carotenoid extraction yields^1^ obtained from papaya (*Carica papaya* L. cv. Sweet Mary) peel tissue applying UAE using soybean oil and ethanol as green extraction solvent, Table S4: Carotenoid extraction yields^1^ obtained from papaya (*Carica papaya* L. cv. Sweet Mary) peel tissue applying UAE using sunflower oil and ethanol as green extraction solvent, Table S5: Physical and physical-chemical characteristics of papaya (*Carica papaya* L. cv. Sweet Mary).

## Sample Availability

Samples of the compounds are not available from the authors.
