# Supplementary material for: Effect of Ultrasound-Assisted Extraction of Carotenoids from Papaya (Carica papaya L. cv. Sweet Mary) Using Vegetable Oils
Source: Molecules. 2022 Jan 19;27(3):638. doi: 10.3390/molecules27030638 (PMC8839964; doi:10.3390/molecules27030638)
Supplement: Supplementary file 1 [file molecules-27-00638-s001.zip › molecules-1542566-supplementary.pdf]

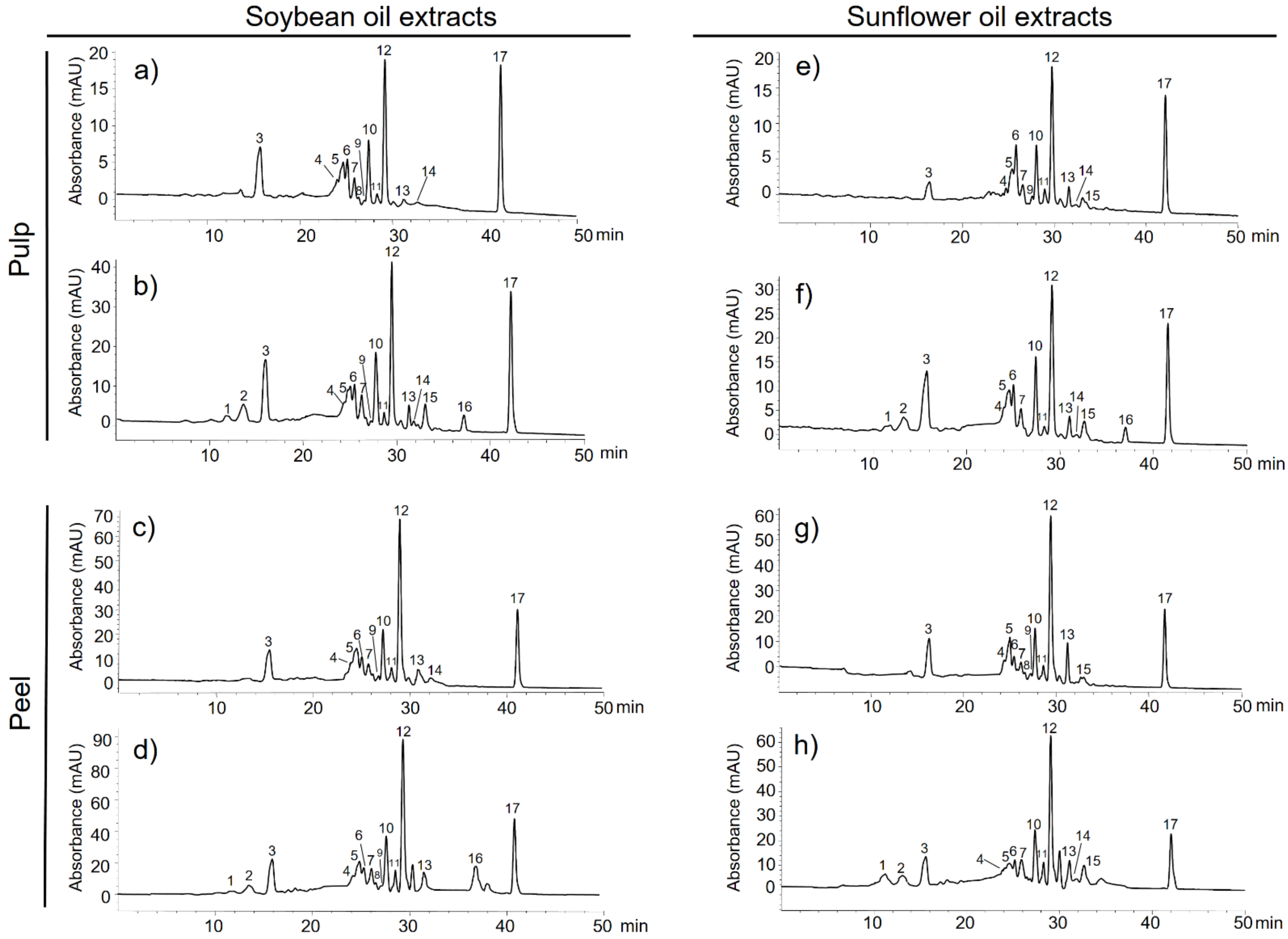

Supplementary Figure S1. C_{30} reversed–phase chromatograms of carotenoids and carotenoid esters identified from papaya (*Carica papaya* L. cv. Sweet Mary) in soybean oil pulp a) control and b) run 9 (60%/ 10 min/ 20% EtOH) extracts and in peel c) control and d) run 14 (60%/ 60 min/ 5% EtOH) extracts; also, in sunflower oil pulp e) control and f) run 9 extracts and in peel g) control and h) run 14 extracts. Peak identities in Table 1.

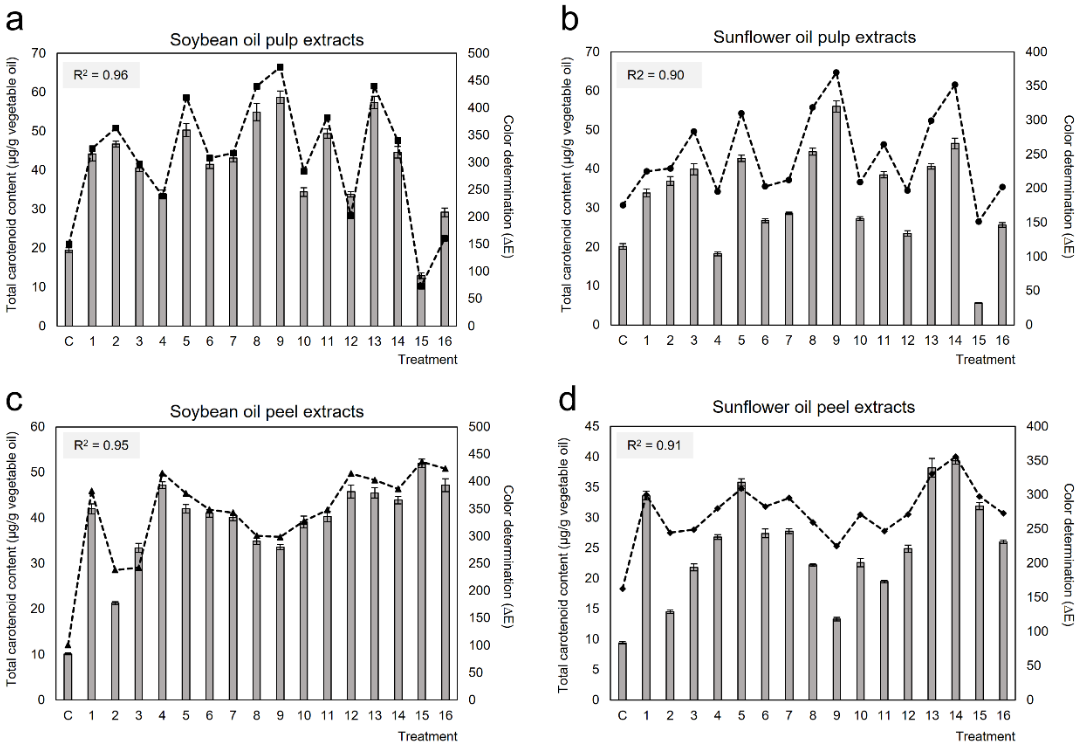

Supplementary Figure S2. Correlation analysis using the regression coefficient (r^2) between total carotenoids content (μg carotenoids/g vegetable oil) and color determination (ΔE) in a) soybean oil and b) sunflower oil pulp extracts and c) soybean oil and d) sunflower oil peel extracts. Letter "C" refers to control and treated samples correspond to the combinations of the variables in the CCD listed in Table 2.

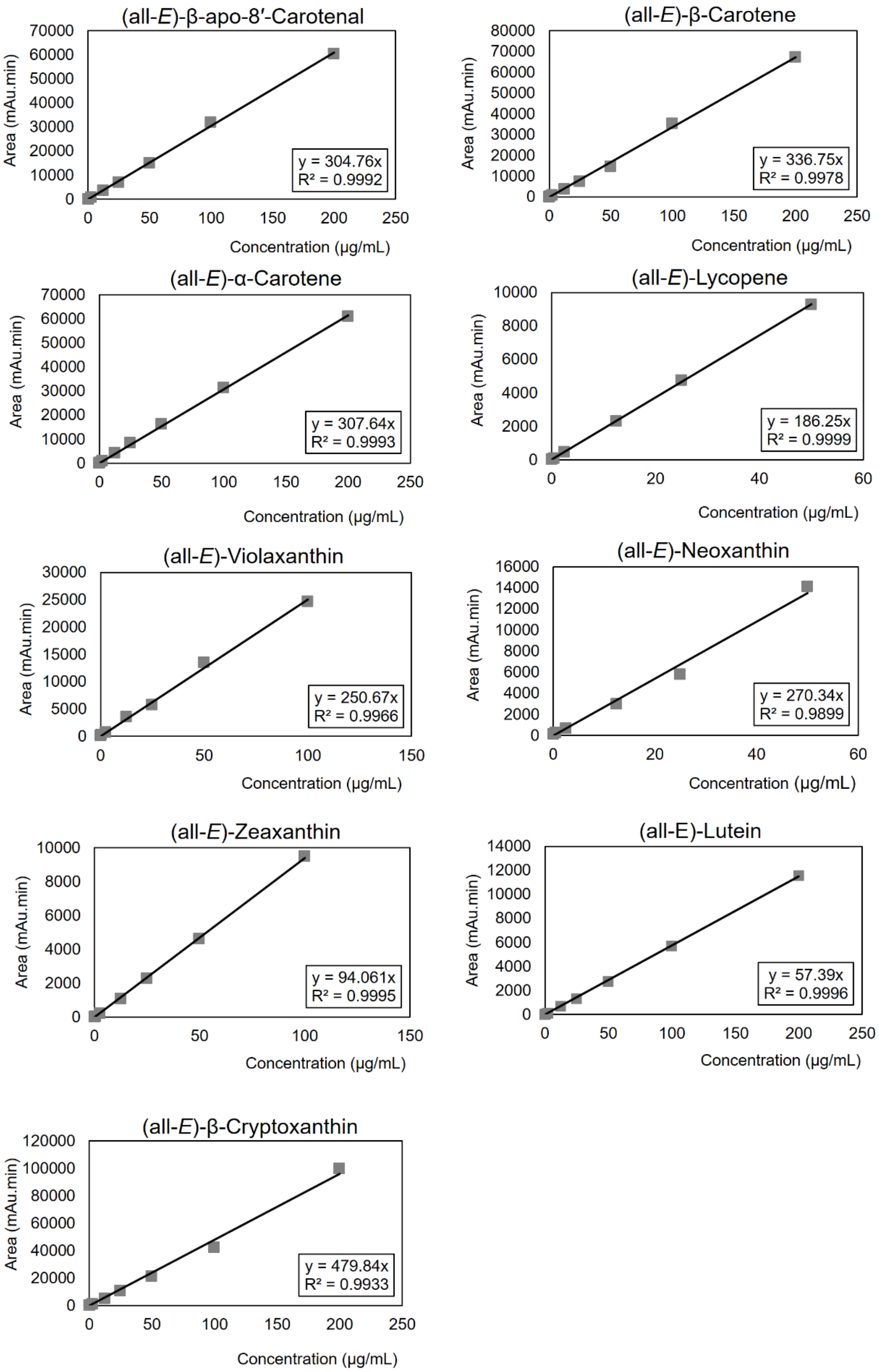

Supplementary Figure S3. Calibration curves of carotenoids standards.

Table S1. Carotenoid extraction yields[1] obtained from papaya (*Carica papaya* L. cv. Sweet Mary) pulp tissue applying UAE using soybean oil and ethanol as green extraction solvent.

**Compound**	**Extraction yield in pulp soybean oil extracts (%)**																
	**C**	**1**	**2**	**3**	**4**	**5**	**6**	**7**	**8**	**9**	**10**	**11**	**12**	**13**	**14**	**15**	**16**
(9Z)-α-cryptoxanthin	0 ± 0^{a}	51 ± 1^{i}	45 ± 2^{h}	34 ± 2^{fg}	24 ± 0^{c}	34 ± 1^{fg}	37 ± 2^{g}	30 ± 2^{ef}	35 ± 1^{g}	34 ± 1^{fg}	30 ± 0^{ef}	27 ± 0^{cde}	25 ± 1^{cd}	43 ± 1^{h}	36 ± 2^{g}	9 ± 0^{b}	29 ± 1^{de}
(all-*E*)-α-cryptoxanthin	0 ± 0^{a}	15 ± 1^{def}	22 ± 0^{i}	15 ± 1^{def}	8 ± 1^{c}	16 ± 1^{fg}	13 ± 1^{de}	15 ± 0^{def}	18 ± 2^{gh}	18 ± 0^{gh}	13 ± 0^{d}	19 ± 0^{h}	16 ± 0^{fg}	20 ± 1^{hi}	16 ± 1^{efg}	4 ± 0^{b}	15 ± 1^{def}
(all-*E*)-β-cryptoxanthin	9 ± 1^{b}	26 ± 0^{fgh}	28 ± 2^{gh}	32 ± 1^{i}	15 ± 2^{c}	24 ± 0^{ef}	21 ± 1^{d}	21 ± 0^{d}	27 ± 1^{fgh}	26 ± 0^{fgh}	18 ± 0^{c}	29 ± 1^{h}	17 ± 1^{c}	23 ± 1^{de}	25 ± 1^{efg}	6 ± 0^{a}	17 ± 0^{c}
(9Z)-violaxanthin laurate	15 ± 0^{ab}	65 ± 5^{ef}	70 ± 1^{fg}	83 ± 2^{hi}	36 ± 1^{d}	75 ± 2^{gh}	58 ± 2^{e}	63 ± 3^{ef}	68 ± 5^{fg}	82 ± 6^{hi}	27 ± 2^{c}	90 ± 4^{i}	38 ± 2^{d}	58 ± 2^{e}	80 ± 0^{h}	9 ± 0^{a}	21 ± 1^{bc}
(all-*E*)-lutein-3-O-myristate	26 ± 0^{d}	36 ± 0^{ef}	45 ± 1^{h}	42 ± 1^{g}	13 ± 1^{b}	26 ± 0^{d}	26 ± 1^{d}	25 ± 0^{d}	37 ± 0^{f}	33 ± 0^{e}	13 ± 0^{b}	37 ± 1^{f}	20 ± 0^{c}	24 ± 1^{d}	34 ± 0^{e}	8 ± 1^{a}	15 ± 0^{b}
(all-*E*)-β-carotene	8 ± 0^{b}	10 ± 0^{cd}	15 ± 0^{i}	14 ± 1^{hi}	8 ± 0^{b}	12 ± 0^{def}	10 ± 0^{cd}	10 ± 0^{c}	12 ± 1^{efg}	13 ± 0^{fgh}	6 ± 0^{b}	14 ± 0^{gh}	7 ± 0^{b}	11 ± 0^{cde}	10 ± 1^{cd}	3 ± 0^{a}	6 ± 0^{b}
(all-*E*)-antheraxanthin myristate palmitate	18 ± 1^{b}	24 ± 0^{de}	49 ± 2^{l}	33 ± 0^{hi}	22 ± 1^{cd}	21 ± 1^{c}	33 ± 1^{hi}	28 ± 1^{fg}	31 ± 1^{gh}	38 ± 1^{k}	26 ± 0^{ef}	37 ± 1^{jk}	21 ± 0^{c}	35 ± 0^{ij}	25 ± 1^{e}	13 ± 1^{a}	26 ± 1^{ef}
(all-*E*)-violaxanthin palmitate	9 ± 2^{bc}	4 ± 0^{a}	11 ± 1^{bc}	11 ± 0^{bc}	6 ± 1^{a}	12 ± 0^{bc}	9 ± 0^{bc}	9 ± 0^{bc}	10 ± 0^{bc}	13 ± 1^{c}	5 ± 0^{a}	10 ± 1^{bc}	4 ± 0^{a}	10 ± 0^{bc}	9 ± 0^{b}	4 ± 0^{a}	5 ± 0^{a}
(9Z)-neoxanthin dibutyrate	13 ± 1^{cde}	9 ± 1^{bcd}	0 ± 0^{a}	10 ± 0^{bcd}	7 ± 1^{bc}	24 ± 1^{f}	11 ± 1^{bcd}	12 ± 1^{cde}	13 ± 0^{de}	17 ± 0^{e}	9 ± 0^{bcd}	14 ± 1^{de}	6 ± 0^{b}	14 ± 1^{de}	14 ± 0^{de}	0 ± 0^{a}	0 ± 0^{a}
(all-*E*)-β-cryptoxanthin caprate	18 ± 1^{b}	32 ± 0^{fgh}	41 ± 3^{i}	29 ± 1^{defg}	20 ± 1^{b}	28 ± 2^{defg}	28 ± 1^{def}	26 ± 0^{cde}	30 ± 2^{efg}	36 ± 1^{h}	25 ± 1^{cd}	33 ± 0^{gh}	23 ± 0^{bc}	25 ± 1^{cd}	30 ± 1^{efg}	8 ± 0^{a}	22 ± 0^{bc}
(all-*E*)-lutein dimyristate	18 ± 0^{a}	40 ± 1^{f}	71 ± 1^{i}	27 ± 0^{bc}	55 ± 3^{h}	31 ± 2^{cde}	35 ± 2^{e}	31 ± 1^{cde}	30 ± 2^{cde}	29 ± 2^{cd}	50 ± 1^{g}	31 ± 1^{cde}	23 ± 1^{b}	23 ± 1^{b}	33 ± 1^{de}	18 ± 1^{a}	48 ± 0^{g}
(all-*E*)-β-cryptoxanthin laurate	29 ± 1^{b}	52 ± 2^{hi}	61 ± 1^{k}	57 ± 2^{jk}	34 ± 1^{c}	53 ± 2^{hi}	48 ± 0^{fg}	44 ± 0^{f}	53 ± 1^{hij}	52 ± 2^{ghi}	38 ± 0^{de}	60 ± 2^{k}	34 ± 0^{cd}	49 ± 2^{gh}	56 ± 1^{ij}	13 ± 1^{a}	40 ± 1^{e}
(all-*E*)-antheraxanthin laurate myristate	18 ± 0^{c}	25 ± 2^{d}	0 ± 0^{a}	23 ± 1^{cd}	9 ± 0^{b}	22 ± 2^{cd}	65 ± 2^{h}	53 ± 1^{g}	29 ± 2^{de}	34 ± 1^{e}	67 ± 1^{h}	28 ± 0^{d}	46 ± 3^{f}	25 ± 1^{d}	27 ± 2^{d}	63 ± 4^{h}	89 ± 3^{i}
(all-*E*)-β-cryptoxanthin myristate	11 ± 1^{b}	30 ± 1^{h}	82 ± 1^{j}	27 ± 1^{gh}	45 ± 2^{i}	23 ± 0^{efg}	25 ± 1^{gh}	18 ± 1^{cdef}	24 ± 2^{fgh}	24 ± 1^{fgh}	18 ± 0^{cde}	24 ± 1^{efgh}	14 ± 0^{bc}	23 ± 1^{def}	25 ± 1^{gh}	4 ± 0^{a}	17 ± 1^{cd}
(13Z)-lycopene isomer 2	0 ± 0^{a}	35 ± 2^{d}	64 ± 2^{i}	34 ± 2^{d}	27 ± 1^{c}	47 ± 1^{f}	46 ± 0^{f}	53 ± 2^{gh}	70 ± 1^{j}	67 ± 1^{ij}	56 ± 1^{h}	46 ± 1^{f}	40 ± 1^{e}	83 ± 1^{l}	77 ± 0^{k}	8 ± 0^{b}	50 ± 2^{fg}
(9Z)-lycopene isomer 4	0 ± 0^{a}	12 ± 1^{b}	44 ± 1^{g}	29 ± 1^{e}	75 ± 2^{k}	24 ± 0^{d}	72 ± 1^{k}	63 ± 1^{i}	67 ± 2^{j}	40 ± 1^{f}	59 ± 0^{i}	23 ± 2^{cd}	49 ± 2^{h}	75 ± 1^{k}	63 ± 2^{i}	14 ± 1^{b}	19 ± 0^{c}
(all-*E*)-lycopene	37 ± 1^{b}	79 ± 1^{g}	46 ± 2^{c}	43 ± 1^{c}	54 ± 1^{d}	94 ± 0^{i}	68 ± 0^{f}	76 ± 2^{g}	78 ± 2^{g}	93 ± 1^{hi}	63 ± 0^{e}	76 ± 2^{g}	55 ± 1^{d}	89 ± 1^{h}	68 ± 1^{f}	14 ± 0^{a}	34 ± 0^{b}
Total hydrocarbon carotenoids	25 ± 1^{b}	46 ± 1^{f}	37 ± 1^{d}	31 ± 1^{c}	38 ± 0^{d}	56 ± 0^{i}	47 ± 0^{f}	50 ± 1^{g}	54 ± 1^{h}	58 ± 1^{j}	42 ± 0^{e}	47 ± 1^{f}	37 ± 0^{d}	60 ± 1^{j}	48 ± 1^{f}	10 ± 0^{a}	24 ± 1^{b}
Total xanthophyll esters	21 ± 0^{b}	35 ± 0^{h}	48 ± 0^{k}	38 ± 0^{i}	25 ± 0^{d}	33 ± 0^{g}	34 ± 0^{g}	31 ± 0^{f}	36 ± 0^{h}	38 ± 0^{i}	27 ± 0^{e}	40 ± 0^{j}	24 ± 0^{c}	31 ± 1^{f}	36 ± 0^{h}	12 ± 0^{a}	27 ± 0^{e}
Total free xanthophylls	9 ± 1^{b}	27 ± 0^{hi}	28 ± 1^{ij}	29 ± 1^{j}	15 ± 1^{c}	24 ± 0^{f}	21 ± 1^{e}	21 ± 0^{e}	26 ± 0^{ghi}	25 ± 0^{fgh}	18 ± 0^{d}	27 ± 1^{hij}	18 ± 1^{d}	24 ± 1^{fg}	24 ± 1^{fg}	6 ± 0^{a}	18 ± 0^{d}
Total xanthophylls (free + esters)	18 ± 0^{b}	33 ± 0^{h}	43 ± 0^{k}	36 ± 1^{j}	22 ± 0^{c}	30 ± 0^{g}	30 ± 0^{g}	28 ± 0^{e}	33 ± 0^{h}	35 ± 0^{i}	24 ± 0^{d}	36 ± 0^{j}	22 ± 0^{c}	29 ± 1^{f}	33 ± 0^{h}	10 ± 0^{a}	25 ± 0^{d}
Total carotenoids	21 ± 0^{b}	38 ± 0^{gh}	40 ± 0^{ij}	34 ± 1^{f}	29 ± 0^{d}	41 ± 0^{jk}	37 ± 0^{g}	38 ± 1^{g}	42 ± 0^{k}	45 ± 0^{l}	32 ± 0^{e}	41 ± 1^{jk}	28 ± 0^{d}	42 ± 1^{k}	39 ± 1^{hi}	10 ± 0^{a}	24 ± 0^{c}
RAE	13 ± 0^{b}	24 ± 1^{fg}	34 ± 1^{i}	28 ± 0^{h}	18 ± 1^{cd}	24 ± 1^{fg}	22 ± 1^{ef}	20 ± 1^{de}	25 ± 1^{gh}	25 ± 1^{gh}	17 ± 1^{c}	27 ± 1^{h}	16 ± 1^{c}	22 ± 1^{ef}	24 ± 0^{fg}	6 ± 0^{a}	16 ± 1^{c}

[1] Carotenoid quantification is accomplished by HPLC-DAD-MS/MS. The numbers correspond to run number listed in the Table 2. Letter "C" refers to control. All results are specified as the mean of two independent determinations ± standard deviation. Results with different alphabets in the same row are significantly different ($p < 0.05$) from each other. Retinol activity equivalents are calculated according to guidelines of the United States (US) Institute of Medicine [41].

Table S2. Carotenoid extraction yields[1] obtained from papaya (*Carica papaya* L. cv. Sweet Mary) pulp tissue applying UAE using sunflower oil and ethanol as green extraction solvent.

**Compound**	**Extraction yield in pulp sunflower oil extracts (%)**																
	**C**	**1**	**2**	**3**	**4**	**5**	**6**	**7**	**8**	**9**	**10**	**11**	**12**	**13**	**14**	**15**	**16**
(9Z)-α-cryptoxanthin	0 ± 0^{a}	40 ± 4^{k}	33 ± 2^{ghij}	37 ± 2^{jk}	22 ± 1^{de}	32 ± 1^{fghi}	0 ± 0^{a}	20 ±1^{c}	31 ± 1^{efghi}	35 ± 0^{hij}	26 ± 2^{de}	27 ± 2^{ef}	27 ± 1^{de}	28 ± 1^{efg}	35 ± 1^{ijk}	9 ± 0^{b}	30 ± 0^{efgh}
(all-*E*)-α-cryptoxanthin	0 ± 0^{a}	16 ± 1^{f}	20 ± 0^{h}	18 ± 0^{gh}	8 ± 0^{c}	12 ± 0^{e}	0 ± 0^{a}	8 ± 0^{cd}	10 ± 0^{d}	12 ± 0^{e}	10 ± 0^{d}	18 ± 0^{g}	16 ± 0^{f}	13 ± 1^{e}	15 ± 0^{f}	3 ± 0^{b}	15 ± 1^{f}
(all-*E*)-β-cryptoxanthin	5 ± 0^{ab}	15 ± 1^{d}	16 ± 1^{d}	34 ± 1^{g}	7 ± 0^{b}	16 ± 0^{d}	6 ± 0^{b}	16 ± 0^{d}	24 ± 1^{e}	25 ± 1^{e}	9 ± 0^{c}	30 ± 1^{f}	10 ± 0^{c}	24 ± 0^{e}	25 ± 1^{e}	3 ± 0^{a}	15 ± 0^{d}
(9Z)-violaxanthin laurate	32 ± 0^{c}	41 ± 0^{d}	32 ± 1^{c}	63 ± 4^{f}	10 ± 0^{b}	48 ± 0^{e}	14 ± 0^{b}	43 ± 2^{de}	71 ± 3^{g}	91 ± 4^{h}	16 ± 1^{b}	93 ± 3^{h}	34 ± 0^{c}	73 ± 0^{g}	0 ± 0^{a}	0 ± 0^{a}	15 ± 0^{b}
(all-*E*)-lutein-3-O-myristate	16 ± 0^{f}	17 ± 1^{f}	8 ± 0^{c}	33 ± 0^{i}	4 ± 0^{b}	27 ± 2^{h}	32 ± 1^{i}	20 ± 0^{g}	36 ± 0^{j}	33 ± 1^{i}	13 ± 0^{d}	33 ± 1^{i}	15 ± 0^{ef}	32 ± 1^{i}	0 ± 0^{a}	0 ± 0^{a}	13 ± 1^{de}
(all-*E*)-β-carotene	11 ± 0^{f}	7 ± 0^{cd}	6 ± 0^{c}	16 ± 0^{h}	2 ± 0^{a}	9 ± 0^{e}	12 ± 0^{g}	8 ± 0^{d}	12 ± 0^{g}	13 ± 0^{g}	4 ± 0^{b}	13 ± 0^{g}	8 ± 0^{d}	11 ± 0^{f}	11 ± 0^{f}	1 ± 0^{a}	7 ± 0^{cd}
(all-*E*)-antheraxanthin myristate palmitate	14 ± 0^{b}	42 ± 0^{k}	30 ± 1^{ij}	22 ± 1^{e}	19 ± 0^{d}	28 ± 0^{ghi}	16 ± 0^{c}	19 ± 0^{d}	25 ± 1^{f}	31 ± 1^{j}	23 ± 0^{ef}	53 ± 0^{l}	12 ± 0^{ab}	27 ± 1^{g}	28 ± 1^{gh}	10 ± 0^{a}	29 ± 1^{hij}
(all-*E*)-violaxanthin palmitate	0 ± 0^{a}	10 ± 0^{g}	6 ± 0^{de}	9 ± 1^{fg}	4 ± 0^{bcd}	7 ± 0^{def}	0 ± 0^{a}	0 ± 0^{a}	9 ± 0^{efg}	10 ± 1^{g}	6 ± 0^{cde}	71 ± 3^{h}	3 ± 0^{ab}	6 ± 0^{de}	7 ± 0^{def}	0 ± 0^{a}	3 ± 0^{bc}
(9Z)-neoxanthin dibutyrate	20 ± 1^{g}	18 ± 0^{f}	8 ± 0^{b}	16 ± 0^{e}	7 ± 0^{b}	8 ± 0^{b}	0 ± 0^{a}	0 ± 0^{a}	0 ± 0^{a}	0 ± 0^{a}	10 ± 1^{c}	17 ± 0^{f}	7 ± 0^{b}	11 ± 1^{d}	11 ± 0^{d}	0 ± 0^{a}	0 ± 0^{a}
(all-*E*)-β-cryptoxanthin caprate	16 ± 1^{bc}	30 ± 2^{fg}	27 ± 1^{f}	31 ± 1^{gh}	15 ± 1^{bc}	24 ± 1^{e}	14 ± 1^{b}	17 ± 0^{bc}	21 ± 1^{de}	27 ± 1^{f}	17 ± 1^{bc}	24 ± 1^{e}	18 ± 1^{cd}	34 ± 1^{h}	28 ± 1^{fg}	6 ± 0^{a}	16 ± 1^{bc}
(all-*E*)-lutein dimyristate	25 ± 0^{cd}	91 ± 4^{i}	53 ± 1^{h}	27 ± 1^{de}	36 ± 1^{f}	27 ± 0^{de}	20 ± 1^{abc}	15 ± 2^{a}	19 ± 1^{ab}	23 ± 1^{bcd}	46 ± 2^{g}	29 ± 0^{e}	16 ± 1^{a}	29 ± 3^{e}	20 ± 1^{ab}	20 ± 0^{abc}	50 ± 2^{gh}
(all-*E*)-β-cryptoxanthin laurate	33 ± 2^{cde}	39 ± 0^{def}	38 ± 1^{def}	62 ± 3^{h}	21 ± 0^{b}	34 ± 1^{cde}	30 ± 2^{c}	33 ± 2^{cd}	44 ± 2^{f}	51 ± 4^{g}	23 ± 0^{b}	43 ± 1^{f}	34 ± 0^{cde}	55 ± 1^{g}	55 ± 2^{g}	6 ± 0^{a}	40 ± 2^{ef}
(all-*E*)-antheraxanthin laurate myristate	21 ± 2^{cd}	49 ± 3^{gh}	50 ± 2^{h}	21 ± 0^{cd}	30 ± 3^{e}	37 ± 0^{f}	25 ± 1^{de}	15 ± 0^{bc}	14 ± 1^{b}	9 ± 0^{b}	42 ± 2^{fg}	22 ± 0^{cd}	42 ± 1^{fg}	22 ± 0^{cd}	24 ± 2^{de}	0 ± 0^{a}	79 ± 3^{i}
(all-*E*)-β-cryptoxanthin myristate	15 ± 1^{ab}	81 ± 4^{g}	27 ± 1^{de}	28 ± 0^{e}	24 ± 2^{de}	27 ± 0^{de}	13 ± 0^{ab}	14 ± 0^{ab}	20 ± 0^{c}	23 ± 1^{cd}	38 ± 2^{f}	25 ± 1^{de}	15 ± 1^{ab}	25 ± 0^{de}	27 ± 0^{de}	11 ± 0^{a}	16 ± 0^{b}
(13Z)-lycopene isomer 2	0 ± 0^{a}	45 ± 0^{g}	83 ± 2^{j}	31 ± 1^{e}	30 ± 3^{e}	59 ± 0^{h}	19 ± 1^{d}	32 ± 0^{e}	32 ± 1^{e}	59 ± 2^{h}	34 ± 1^{e}	40 ± 1^{f}	22 ± 1^{d}	57 ± 1^{h}	79 ± 2^{i}	7 ± 0^{b}	13 ± 0^{c}
(9Z)-lycopene isomer 4	0 ± 0^{a}	40 ± 2^{g}	77 ± 1^{k}	14 ± 1^{c}	22 ± 1^{d}	50 ± 2^{j}	13 ± 1^{c}	27 ± 0^{e}	26 ± 1^{e}	39 ± 2^{g}	45 ± 0^{hi}	19 ± 0^{d}	34 ± 1^{f}	44 ± 1^{h}	48 ± 1^{ij}	7 ± 0^{b}	20 ± 0^{d}
(all-*E*)-lycopene	29 ± 1^{bc}	39 ± 0^{e}	49 ± 1^{f}	40 ± 0^{e}	32 ± 1^{cd}	77 ± 1^{h}	50 ± 1^{f}	57 ± 1^{g}	76 ± 2^{h}	95 ± 4^{i}	52 ± 1^{f}	33 ± 0^{d}	28 ± 0^{bc}	48 ± 0^{f}	82 ± 1^{i}	3 ± 0^{a}	28 ± 0^{b}
Total hydrocarbon carotenoids	21 ± 1^{b}	29 ± 0^{d}	39 ± 1^{g}	29 ± 0^{d}	21 ± 1^{b}	50 ± 1^{i}	31 ± 1^{e}	36 ± 1^{f}	47 ± 1^{h}	59 ± 2^{k}	34 ± 0^{ef}	25 ± 1^{c}	21 ± 0^{b}	35 ± 0^{f}	54 ± 1^{j}	3 ± 0^{a}	19 ± 0^{b}
Total xanthophyll esters	20 ± 0^{c}	38 ± 0^{k}	27 ± 0^{g}	36 ± 0^{j}	16 ± 0^{b}	28 ± 1^{g}	21 ± 0^{cd}	20 ± 0^{cd}	30 ± 0^{h}	34 ± 1^{i}	21 ± 0^{d}	40 ± 1^{l}	20 ± 0^{c}	35 ± 0^{ij}	24 ± 0^{e}	6 ± 0^{a}	26 ± 0^{f}
Total free xanthophylls	5 ± 0^{a}	18 ± 0^{f}	19 ± 1^{f}	32 ± 1^{j}	8 ± 0^{b}	17 ± 0^{ef}	5 ± 0^{a}	15 ± 0^{e}	22 ± 1^{g}	24 ± 1^{h}	11 ± 0^{c}	28 ± 1^{i}	13 ± 0^{d}	22 ± 0^{gh}	24 ± 1^{h}	4 ± 0^{a}	17 ± 0^{ef}
Total xanthophylls (free + esters)	17 ± 0^{c}	32 ± 0^{h}	25 ± 1^{f}	35 ± 0^{i}	14 ± 0^{b}	25 ± 1^{f}	16 ± 0^{c}	19 ± 0^{d}	28 ± 0^{g}	31 ± 1^{h}	19 ± 0^{d}	37 ± 1^{j}	18 ± 0^{d}	32 ± 0^{h}	24 ± 0^{ef}	5 ± 0^{a}	23 ± 0^{e}
Total carotenoids	19 ± 0^{bc}	31 ± 0^{f}	31 ± 1^{f}	32 ± 0^{fg}	17 ± 1^{b}	35 ± 1^{h}	23 ± 1^{d}	26 ± 0^{e}	36 ± 0^{h}	43 ± 2^{i}	25 ± 0^{e}	32 ± 1^{fg}	19 ± 0^{c}	33 ± 0^{g}	37 ± 0^{h}	4 ± 0^{a}	21 ± 0^{d}
RAE	14 ± 0^{d}	22 ± 1^{fg}	18 ± 0^{e}	30 ± 1^{i}	9 ± 0^{b}	18 ± 0^{e}	15 ± 1^{d}	15 ± 0^{d}	21 ± 1^{f}	24 ± 0^{h}	12 ± 0^{c}	24 ± 0^{gh}	15 ± 1^{d}	24 ± 1^{h}	24 ± 1^{h}	4 ± 0^{a}	16 ± 1^{d}

[1] Carotenoid quantification is accomplished by HPLC-DAD-MS/MS. The numbers correspond to run number listed in the Table 2. Letter "C" refers to control. All results are specified as the mean of two independent determinations ± standard deviation. Results with different alphabets in the same row are significantly different ($p < 0.05$) from each other. Retinol activity equivalents (RAE) are calculated (µg carotenoids/100 g fresh weight) according to guidelines of the United States (US) Institute of Medicine [41].

Table S3. Carotenoid extraction yields[1] obtained from papaya (*Carica papaya* L. cv. Sweet Mary) peel tissue applying UAE using soybean oil and ethanol as green extraction solvent.

Compound	Extraction yield in peel soybean oil extracts (%)																
	C	1	2	3	4	5	6	7	8	9	10	11	12	13	14	15	16
(9Z)-α-cryptoxanthin	0 ± 0[a]	57 ± 3[d]	54 ± 1[d]	66 ± 2[ef]	75 ± 1[g]	68 ± 2[ef]	66 ± 3[ef]	81 ± 0[h]	65 ± 1[e]	58 ± 1[d]	56 ± 1[d]	71 ± 1[fg]	38 ± 0[c]	29 ± 1[b]	65 ± 1[ef]	74 ± 3[g]	41 ± 2[c]
(all-*E*)-α-cryptoxanthin	0 ± 0[a]	24 ± 1[g]	21 ± 0[f]	18 ± 0[d]	26 ± 0[h]	19 ± 0[de]	18 ± 0[d]	23 ± 1[g]	21 ± 1[f]	14 ± 1[c]	15 ± 0[c]	20 ± 0[ef]	19 ± 1[de]	12 ± 0[b]	24 ± 0[g]	27 ± 0[h]	24 ± 1[g]
(all-*E*)-β-cryptoxanthin	13 ± 0[a]	52 ± 1[g]	19 ± 0[b]	44 ± 0[de]	47 ± 1[ef]	47 ± 0[ef]	44 ± 1[de]	37 ± 1[c]	42 ± 2[d]	41 ± 2[d]	36 ± 1[c]	45 ± 0[def]	44 ± 0[de]	44 ± 1[de]	48 ± 0[f]	46 ± 0[ef]	43 ± 2[de]
(9Z)-violaxanthin laurate	18 ± 1[a]	58 ± 2[e]	18 ± 1[a]	33 ± 1[b]	46 ± 1[d]	49 ± 2[d]	62 ± 0[ef]	50 ± 1[d]	40 ± 0[c]	62 ± 2[ef]	49 ± 0[d]	70 ± 2[gh]	65 ± 1[f]	66 ± 1[fg]	71 ± 2[h]	62 ± 0[ef]	38 ± 2[c]
(all-*E*)-lutein-3-O-myristate	14 ± 0[a]	54 ± 2[g]	14 ± 1[a]	31 ± 1[b]	49 ± 2[efg]	30 ± 2[b]	45 ± 1[de]	39 ± 1[c]	19 ± 1[a]	38 ± 2[c]	38 ± 1[c]	47 ± 1[ef]	48 ± 3[ef]	55 ± 2[g]	50 ± 1[efg]	51 ± 1[fg]	40 ± 2[cd]
(all-*E*)-β-carotene	7 ± 0[a]	26 ± 0[g]	13 ± 0[b]	21 ± 0[de]	26 ± 0[g]	23 ± 0[f]	21 ± 1[de]	19 ± 0[c]	20 ± 1[cde]	19 ± 1[cde]	19 ± 0[cd]	21 ± 0[e]	23 ± 0[f]	19 ± 0[cd]	20 ± 0[cde]	24 ± 0[f]	27 ± 1[g]
(all-*E*)-antheraxanthin myristate palmitate	12 ± 0[a]	46 ± 0[cd]	15 ± 0[a]	36 ± 0[b]	73 ± 5[h]	42 ± 1[bc]	47 ± 1[cde]	47 ± 2[cde]	51 ± 1[def]	41 ± 1[bc]	61 ± 0[g]	41 ± 2[bc]	56 ± 4[fg]	53 ± 2[ef]	45 ± 2[cd]	72 ± 2[h]	81 ± 1[i]
(all-*E*)-violaxanthin palmitate	5 ± 0[a]	18 ± 0[g]	9 ± 0[b]	13 ± 0[cd]	22 ± 2[h]	15 ± 0[de]	17 ± 1[efg]	13 ± 1[c]	16 ± 0[efg]	13 ± 1[cd]	16 ± 0[ef]	16 ± 0[efg]	16 ± 0[ef]	15 ± 1[ef]	17 ± 0[fg]	23 ± 0[h]	33 ± 1[i]
(9Z)-neoxanthin dibutyrate	11 ± 0[a]	39 ± 0[de]	9 ± 0[a]	26 ± 1[b]	46 ± 0[f]	37 ± 1[d]	31 ± 1[c]	31 ± 0[c]	35 ± 0[d]	23 ± 0[b]	36 ± 1[d]	32 ± 2[c]	44 ± 3[f]	42 ± 1[ef]	42 ± 0[ef]	50 ± 1[g]	53 ± 1[g]
(all-*E*)-β-cryptoxanthin caprate	11 ± 0[a]	36 ± 1[de]	23 ± 1[b]	33 ± 1[c]	43 ± 1[g]	36 ± 1[de]	39 ± 0[ef]	38 ± 0[de]	38 ± 0[de]	33 ± 1[c]	43 ± 1[g]	35 ±1[cd]	41 ± 0[fg]	38 ± 1[de]	38 ± 1[de]	46 ± 1[h]	49 ± 2[i]
(all-*E*)-lutein dimyristate	14 ± 0[a]	45 ± 1[efg]	49 ± 2[g]	35 ± 2[bc]	62 ± 1[h]	44 ± 2[efg]	42 ± 1[def]	38 ± 1[bcd]	38 ± 2[bcd]	35 ± 2[bc]	48 ± 1[g]	33 ± 1[b]	45 ± 1[fg]	44 ± 0[efg]	40 ± 1[cde]	66 ± 2[h]	72 ± 3[i]
(all-*E*)-β-cryptoxanthin laurate	22 ± 0[a]	88 ± 2[k]	37 ± 0[b]	71 ± 0[de]	85 ± 0[jk]	79 ± 2[hi]	74 ± 1[efg]	66 ± 1[c]	71 ± 1[def]	68 ± 1[cd]	65 ± 1[c]	74 ± 1[efg]	72 ± 1[efg]	76 ± 3[gh]	82 ± 0[ij]	80 ± 0[i]	75 ± 1[fg]
(all-*E*)-antheraxanthin laurate myristate	5 ± 0[a]	17 ± 0[cde]	73 ± 1[l]	13 ± 1[b]	48 ± 0[j]	18 ± 0[ef]	18 ± 1[f]	18 ± 1[f]	15 ± 0[c]	13 ± 0[b]	25 ± 0[h]	16 ± 1[cd]	22 ± 0[g]	17 ± 1[def]	16 ± 1[cde]	59 ± 0[k]	41 ± 1[i]
(all-*E*)-β-cryptoxanthin myristate	8 ± 0[a]	25 ± 0[fg]	15 ± 0[b]	21 ± 1[c]	30 ± 0[k]	27 ± 1[ij]	23 ± 0[d]	21 ± 1[c]	21 ± 0[c]	20 ± 1[c]	21 ± 0[c]	24 ± 0[ef]	23 ± 0[de]	25 ± 0[fg]	28 ± 0[j]	27 ± 0[hi]	26 ± 0[gh]
(13Z)-lycopene isomer 2	0 ± 0[a]	57 ± 2[g]	33 ± 0[b]	33 ± 0[b]	49 ± 2[de]	71 ± 1[h]	70 ± 1[h]	48 ± 2[d]	42 ± 1[c]	41 ± 1[c]	41 ± 1[c]	52 ± 1[ef]	75 ± 3[i]	53 ± 1[efg]	46 ± 0[d]	54 ± 1[fg]	41 ± 1[c]
(9Z)-lycopene isomer 4	0 ± 0[a]	43 ± 0[ef]	24 ± 0[b]	27 ± 0[b]	38 ± 1[cd]	65 ± 2[j]	63 ± 1[j]	54 ± 1[h]	45 ± 1[f]	36 ± 1[c]	41 ± 3[de]	58 ± 2[i]	66 ± 2[j]	44 ± 1[ef]	57 ± 0[hi]	50 ± 1[g]	54 ± 0[h]
(all-*E*)-lycopene	19 ± 0[a]	65 ± 0[e]	28 ± 0[b]	54 ± 1[c]	70 ± 1[f]	79 ± 1[hi]	74 ± 2[g]	70 ± 1[f]	72 ± 1[fg]	61 ± 1[d]	60 ± 0[d]	59 ± 1[d]	78 ± 0[h]	81 ± 2[i]	74 ± 1[g]	64 ± 1[e]	67 ± 1[e]
Total hydrocarbon carotenoids	15 ± 0[a]	50 ± 0[e]	23 ± 0[b]	41 ± 1[c]	53 ± 0[fg]	60 ± 0[k]	56 ± 1[hi]	51 ± 1[ef]	53 ± 0[fg]	45 ± 1[d]	45 ± 0[d]	46 ± 1[d]	59 ± 0[jk]	58 ± 1[ij]	54 ± 1[gh]	50 ± 0[e]	52 ± 1[f]
Total xanthophyll esters	13 ± 0[a]	48 ± 0[j]	28 ± 0[b]	36 ± 1[c]	54 ± 0[l]	42 ± 1[f]	44 ± 0[h]	39 ± 0[e]	37 ± 0[d]	39 ± 1[e]	43 ± 0[gh]	43 ± 0[g]	46 ± 0[i]	47 ± 0[ij]	47 ± 0[ij]	56 ± 0[m]	53 ± 0[k]
Total free xanthophylls	13 ± 0[a]	47 ± 1[l]	22 ± 0[b]	41 ± 0[ghi]	45 ± 0[kl]	43 ± 0[ijk]	41 ± 0[fgh]	38 ± 0[def]	40 ± 1[efgh]	37 ± 2[de]	33 ± 1[c]	42 ± 0[hij]	39 ± 0[defg]	36 ± 1[d]	45 ± 0[jkl]	45 ± 1[jkl]	40 ± 2[efgh]
Total xanthophylls (free + esters)	13 ± 0[a]	48 ± 0[l]	27 ± 0[b]	36 ± 0[c]	53 ± 0[k]	42 ± 1[f]	43 ± 0[g]	39 ± 0[e]	38 ± 0[d]	38 ± 1[de]	42 ± 0[f]	43 ± 0[fg]	45 ± 0[h]	46 ± 0[h]	47 ± 0[i]	55 ± 0[l]	51 ± 1[j]
Total carotenoids	14 ± 0[a]	48 ± 0[h]	26 ± 0[b]	38 ± 0[c]	53 ± 0[j]	47 ± 0[g]	47 ± 0[g]	43 ± 0[ef]	42 ± 0[e]	40 ± 1[d]	43 ± 0[ef]	44 ± 0[f]	49 ± 0[h]	49 ± 1[h]	49 ± 0[h]	53 ± 0[j]	51 ± 1[i]
RAE	8 ± 0[a]	30 ± 0[hi]	14 ± 0[b]	25 ± 0[d]	31 ± 0[i]	28 ± 0[g]	26 ± 0[ef]	23 ± 0[c]	25 ± 0[de]	24 ± 1[c]	24 ± 0[c]	26 ± 0[ef]	27 ± 0[f]	26 ± 0[ef]	28 ± 0[g]	29 ± 0[gh]	29 ± 1[gh]

[1] Carotenoid quantification is accomplished by HPLC-DAD-MS/MS. The numbers correspond to run number listed in the Table 2. Letter "C" refers to control. All results are specified as the mean of two independent determinations ± standard deviation. Results with different alphabets in the same row are significantly different ($p < 0.05$) from each other. Retinol activity equivalents (RAE) are calculated (μg carotenoids/100 g fresh weight) according to guidelines of the United States (US) Institute of Medicine [41].

Table S4. Carotenoid extraction yields[1] obtained from papaya (*Carica papaya* L. cv. Sweet Mary) peel tissue applying UAE using sunflower oil and ethanol as green extraction solvent..

Compound	Extraction yield in peel sunflower oil extracts (%)																
	**C**	**1**	**2**	**3**	**4**	**5**	**6**	**7**	**8**	**9**	**10**	**11**	**12**	**13**	**14**	**15**	**16**
(9Z)-α-cryptoxanthin	0 ± 0^{a}	44 ± 1^{f}	26 ± 1^{b}	0 ± 0^{a}	42 ± 1^{e}	63 ± 1^{h}	31 ± 1^{c}	58 ± 1^{g}	67 ± 1^{i}	0 ± 0^{a}	0 ± 0^{a}	0 ± 0^{a}	0 ± 0^{a}	0 ± 0^{a}	57 ± 2^{g}	58 ± 1^{g}	35 ± 1^{d}
(all-*E*)-α-cryptoxanthin	0 ± 0^{a}	14 ± 0^{d}	17 ± 0^{e}	0 ± 0^{a}	12 ± 1^{c}	19 ± 0^{g}	11 ± 0^{b}	19 ± 0^{g}	18 ± 0^{ef}	0 ± 0^{a}	0 ± 0^{a}	0 ± 0^{a}	0 ± 0^{a}	0 ± 0^{a}	25 ± 0^{i}	21 ± 0^{h}	19 ± 1^{fg}
(all-*E*)-β-cryptoxanthin	13 ± 0^{b}	47 ± 0^{j}	19 ± 0^{d}	29 ± 0^{f}	17 ± 0^{cd}	40 ± 0^{i}	39 ± 0^{i}	32 ± 0^{g}	34 ± 0^{h}	11 ± 0^{a}	19 ± 0^{d}	16 ± 1^{c}	17 ± 1^{cd}	29 ± 2^{ef}	45 ± 1^{j}	27 ± 0^{e}	17 ± 1^{cd}
(9Z)-violaxanthin laurate	10 ± 0^{b}	0 ± 0^{a}	0 ± 0^{a}	0 ± 0^{a}	0 ± 0^{a}	0 ± 0^{a}	0 ± 0^{a}	0 ± 0^{a}	0 ± 0^{a}	0 ± 0^{a}	16 ± 0^{c}	0 ± 0^{a}	22 ± 1^{d}	62 ± 1^{g}	45 ± 2^{f}	32 ± 0^{e}	0 ± 0^{a}
(all-*E*)-lutein-3-O-myristate	9 ± 0^{b}	0 ± 0^{a}	0 ± 0^{a}	0 ± 0^{a}	0 ± 0^{a}	0 ± 0^{a}	0 ± 0^{a}	0 ± 0^{a}	0 ± 0^{a}	0 ± 0^{a}	36 ± 1^{d}	0 ± 0^{a}	23 ± 1^{c}	57 ± 1^{e}	34 ± 2^{d}	23 ± 1^{c}	0 ± 0^{a}
(all-*E*)-β-carotene	7 ± 0^{b}	21 ± 0^{i}	7 ± 0^{b}	14 ± 0^{d}	3 ± 0^{a}	20 ± 0^{h}	18 ± 0^{g}	15 ± 1^{de}	16 ± 1^{ef}	7 ± 0^{b}	11 ± 0^{c}	10 ± 0^{c}	17 ± 0^{fg}	25 ± 0^{k}	23 ± 0^{j}	14 ± 0^{d}	6 ± 0^{b}
(all-*E*)-antheraxanthin myristate palmitate	6 ± 0^{a}	20 ± 2^{b}	26 ± 2^{c}	28 ±2^{cd}	28 ± 1^{cd}	45 ± 0^{g}	41 ± 2^{fg}	38 ± 1^{ef}	33 ± 2^{de}	17 ± 1^{b}	44 ± 2^{g}	27 ± 1^{c}	42 ± 2^{fg}	45 ± 3^{g}	20 ± 0^{b}	55 ± 1^{h}	29 ± 1^{cd}
(all-*E*)-violaxanthin palmitate	4 ± 0^{b}	13 ± 0^{ef}	4 ± 0^{b}	9 ± 0^{c}	5 ± 0^{b}	15 ± 1^{f}	10 ± 0^{d}	15 ± 1^{f}	12 ± 0^{e}	4 ± 0^{b}	0 ± 0^{a}	4 ± 0^{b}	3 ± 0^{b}	27 ± 1^{h}	19 ± 0^{g}	12 ± 0^{de}	0 ± 0^{a}
(9Z)-neoxanthin dibutyrate	11 ± 0^{d}	29 ± 1^{h}	8 ± 0^{c}	20 ± 0^{ef}	10 ± 0^{cd}	32 ± 1^{i}	27 ± 1^{gh}	27 ± 0^{gh}	22 ± 1^{f}	10 ± 0^{cd}	0 ± 1^{a}	8 ± 0^{c}	5 ± 0^{b}	88 ± 1^{j}	18 ± 0^{e}	25 ± 2^{g}	0 ± 0^{a}
(all-*E*)-β-cryptoxanthin caprate	12 ± 0^{a}	36 ± 0^{k}	20 ± 0^{ef}	24 ± 1^{g}	19 ± 1^{def}	41 ± 1^{l}	34 ± 1^{jk}	36 ± 0^{k}	30 ± 1^{h}	14 ± 0^{b}	20 ± 0^{f}	17 ± 1^{cd}	18 ± 0^{cde}	33 ± 2^{ij}	44 ± 1^{m}	32 ± 0^{hi}	16 ± 0^{bc}
(all-*E*)-lutein dimyristate	14 ± 1^{a}	40 ± 2^{g}	24 ± 0^{c}	28 ± 1^{d}	47 ± 0^{h}	44 ± 1^{h}	34 ± 2^{ef}	37 ± 0^{fg}	33 ± 2^{e}	19 ± 1^{b}	52 ± 0^{i}	39 ± 1^{g}	66 ± 2^{j}	80 ± 1^{k}	53 ± 1^{i}	45 ± 0^{h}	33 ± 1^{e}
(all-*E*)-β-cryptoxanthin laurate	24 ± 0^{a}	76 ± 1^{j}	35 ± 1^{c}	55 ± 0^{f}	33 ± 0^{c}	70 ± 1^{i}	51 ± 0^{e}	57 ± 0^{g}	56 ± 1^{fg}	29 ± 0^{b}	36 ± 1^{d}	35 ± 0^{cd}	37 ± 1^{d}	66 ± 0^{h}	83 ± 0^{k}	50 ± 1^{e}	33 ± 0^{c}
(all-*E*)-antheraxanthin laurate myristate	5 ± 0^{a}	15 ± 1^{c}	23 ± 1^{e}	11 ± 0^{b}	73 ± 0^{k}	19 ± 0^{d}	15 ± 0^{c}	16 ± 0^{c}	15 ± 1^{c}	12 ± 0^{b}	66 ± 2^{j}	28 ± 0^{f}	17 ± 1^{l}	42 ± 1^{g}	24 ± 0^{e}	45 ± 1^{h}	51 ± 1^{i}
(all-*E*)-β-cryptoxanthin myristate	8 ± 0^{a}	26 ± 0^{ef}	12 ± 1^{b}	16 ± ^{cd}	14 ± 0^{c}	26 ± 1^{ef}	17 ± 1^{cd}	19 ± 1^{d}	19 ± 1^{d}	16 ± 1^{cd}	24 ± 2^{e}	17 ± 0^{cd}	28 ± 1^{f}	37 ± 1^{h}	32 ± 0^{g}	18 ± 0^{d}	16 ± 0^{cd}
(13Z)-lycopene isomer 2	0 ± 0^{a}	53 ± 1^{gh}	30 ± 1^{cd}	39 ± 0^{e}	61 ± 2^{hi}	43 ± 2^{ef}	42 ± 2^{ef}	49 ± 3^{fg}	51 ± 2^{fg}	45 ± 0^{efg}	37 ± 0^{de}	20 ± 0^{b}	26 ± 3^{bc}	26 ± 1^{bc}	67 ± 4^{i}	41 ± 1^{k}	75 ± 0^{j}
(9Z)-lycopene isomer 4	0 ± 0^{a}	43 ± 3^{e}	16 ± 1^{b}	0 ± 0^{a}	55 ± 1^{h}	44 ± 1^{ef}	51 ± 2^{fgh}	45 ± 3^{efg}	31 ± 1^{cd}	25 ± 1^{c}	47 ± 0^{efg}	35 ± 1^{d}	25 ± 2^{c}	31 ± 2^{cd}	52 ± 4^{gh}	29 ± 0^{j}	77 ± 1^{i}
(all-*E*)-lycopene	20 ± 0^{a}	68 ± 0^{i}	25 ± 1^{b}	46 ± 0^{e}	71 ± 1^{i}	68 ± 0^{i}	53 ± 2^{h}	50 ± 2^{fg}	47 ± 0^{ef}	18 ± 0^{a}	28 ± 1^{c}	23 ± 0^{b}	18 ± 0^{a}	51 ± 2^{gh}	82 ± 0^{k}	36 ± 1^{d}	75 ± 1^{j}
Total hydrocarbon carotenoids	15 ± 0^{a}	51 ± 0^{hi}	18 ± 0^{b}	33 ± 0^{d}	47 ± 1^{g}	50 ± 0^{h}	41 ± 1^{f}	38 ± 1^{e}	36 ± 0^{e}	16 ± 0^{a}	24 ± 1^{c}	20 ± 0^{b}	19 ± 0^{b}	40 ± 2^{f}	60 ± 0^{j}	28 ± 1^{e}	53 ± 0^{i}
Total xanthophyll esters	12 ± 0^{a}	30 ± 0^{i}	17 ± 0^{c}	22 ± 0^{e}	24 ± 0^{f}	32 ± 1^{j}	25 ± 0^{g}	27 ± 0^{h}	25 ± 0^{g}	14 ± 0^{b}	33 ± 0^{k}	19 ± 0^{d}	37 ± 0^{m}	53 ± 0^{o}	43 ± 0^{n}	35 ± 0^{l}	20 ± 0^{d}
Total free xanthophylls	13 ± 0^{c}	40 ± 1^{j}	19 ± 0^{d}	21 ± 0^{e}	18 ± 0^{d}	38 ± 0^{i}	33 ± 0^{gh}	31 ± 0^{g}	33 ± 0^{h}	8 ± 0^{a}	14 ± 0^{c}	11 ± 0^{b}	13 ± 1^{bc}	21 ± 1^{e}	42 ± 0^{k}	28 ± 0^{f}	19 ± 0^{d}
Total xanthophylls (free + esters)	12 ± 0^{a}	31 ± 0^{j}	17 ± 0^{c}	22 ± 0^{f}	23 ± 0^{g}	33 ± 0^{k}	26 ± 0^{h}	28 ± 0^{i}	26 ± 0^{h}	13 ± 0^{b}	31 ± 0^{j}	18 ± 0^{d}	33 ± 0^{k}	49 ± 1^{n}	43 ± 0^{m}	35 ± 0^{l}	20 ± 0^{e}
Total carotenoids	13 ± 0^{a}	37 ± 0^{h}	18 ± 0^{b}	25 ± 0^{d}	30 ± 0^{f}	38 ± 0^{h}	30 ± 0^{f}	30 ± 1^{f}	29 ± 0^{e}	14 ± 0^{a}	29 ± 0^{e}	19 ± 0^{c}	29 ± 0^{ef}	47 ± 1^{i}	48 ± 0^{j}	33 ± 0^{g}	29 ± 0^{ef}
RAE	8 ± 0^{a}	27 ± 0^{j}	12 ± 0^{c}	18 ± 0^{g}	11 ± 0^{bc}	26 ± 0^{i}	21 ± 0^{h}	20 ± 0^{h}	20 ± 0^{h}	11 ± 0^{b}	15 ± 1^{e}	13 ± 0^{d}	17 ± 0^{f}	27 ± 0^{j}	30 ± 0^{k}	18 ± 0^{g}	12 ± 0^{c}

[1] Carotenoid quantification is accomplished by HPLC-DAD-MS/MS. The numbers correspond to run number listed in the Table 2. Letter "C" refers to control. All results are specified as the mean of two independent determinations ± standard deviation. Results with different alphabets in the same row are significantly different ($p < 0.05$) from each other. Retinol activity equivalents (RAE) are calculated (µg carotenoids/100 g fresh weight) according to guidelines of the United States (US) Institute of Medicine [41].

Table S5. Physical and physical-chemical characteristics of papaya (*Carica papaya* L. cv. Sweet Mary).

**Characteristic**	**Value**[1]
Total weight of whole fruit (g)	1128.9 ± 165.6
Apical calibre (cm)	20.9 ± 0.6
Equatorial calibre (cm)	9.5 ± 0.2
Titratable acidity (g citric acid/100 g fresh weight)	0.12 ± 0.00
pH	5.2 ± 0.1
Soluble solids (°Brix at 25 °C)	10.2 ± 0.3
Moisture content (% wet basis)	84.9 ± 1.7
Pulp color parameters	
L*	62.4 ± 10.0
a*	22.4 ± 7.1
b*	26.8 ± 5.1
Peel color parameters	
L*	52.6 ± 5.4
a*	13.0 ± 1.2
b*	21.4 ± 5.1

[1] Values are the mean of three independent determinations ±standard deviation.
